# BAG3 Proteomic Signature under Proteostasis Stress

**DOI:** 10.3390/cells9112416

**Published:** 2020-11-04

**Authors:** Christof Hiebel, Elisabeth Stürner, Meike Hoffmeister, Georg Tascher, Mario Schwarz, Heike Nagel, Christian Behrends, Christian Münch, Christian Behl

**Affiliations:** 1 Institute of Pathobiochemistry, The Autophagy Lab, University Medical Center of the Johannes Gutenberg University, Duesbergweg 6, 55128 Mainz, Germany; christof.hiebel@gmail.com (C.H.); stuerner.elisabeth@gmx.de (E.S.); marschwa@uni-mainz.de (M.S.); nagelh@uni-mainz.de (H.N.); 2 Institute of Biochemistry II, Faculty of Medicine, Goethe University, Theodor-Stern-Kai 7, 60590 Frankfurt am Main, Germany; Meike.Hoffmeister@mhb-fontane.de (M.H.); tascher@med.uni-frankfurt.de (G.T.); Christian.Behrends@mail03.med.uni-muenchen.de (C.B.); muench@biochem2.de (C.M.)

**Keywords:** BAG3, proteostasis, protein quality control, stress response, autophagy, interactome

## Abstract

The multifunctional HSP70 co-chaperone BAG3 (BCL-2-associated athanogene 3) represents a key player in the quality control of the cellular proteostasis network. In response to stress, BAG3 specifically targets aggregation-prone proteins to the perinuclear aggresome and promotes their degradation via BAG3-mediated selective macroautophagy. To adapt cellular homeostasis to stress, BAG3 modulates and functions in various cellular processes and signaling pathways. Noteworthy, dysfunction and deregulation of BAG3 and its pathway are pathophysiologically linked to myopathies, cancer, and neurodegenerative disorders. Here, we report a BAG3 proteomic signature under proteostasis stress. To elucidate the dynamic and multifunctional action of BAG3 in response to stress, we established BAG3 interactomes under basal and proteostasis stress conditions by employing affinity purification combined with quantitative mass spectrometry. In addition to the identification of novel potential BAG3 interactors, we defined proteins whose interaction with BAG3 was altered upon stress. By functional annotation and protein-protein interaction enrichment analysis of the identified potential BAG3 interactors, we confirmed the multifunctionality of BAG3 and highlighted its crucial role in diverse cellular signaling pathways and processes, ensuring cellular proteostasis and cell viability. These include protein folding and degradation, gene expression, cytoskeleton dynamics (including cell cycle and transport), as well as granulostasis, in particular.

## 1. Introduction

In eukaryotic cells, autophagy and the ubiquitin–proteasome system represent the two principal protein quality control systems ensuring cellular homeostasis [1,2,3,4,5,6]. To that end, both processes closely collaborate with molecular chaperone systems, such as the HSP70 (*heat shock protein 70*) system and its co-chaperones [7,8,9,10,11]. The HSP70 co-chaperone BAG3 (*BCL-2-associated anthanogene 3*), also named BIS (*BCL-2 interacting death suppressor*) or CAIR-1, belongs to the highly conserved family of BAG co-chaperones and plays a pivotal role in this cellular proteostasis network [12,13,14,15,16,17,18].

During cell aging and under acute proteotoxic stress, we discovered a reciprocally regulated expression of the BAG proteins BAG1 and BAG3 (BAG1/BAG3 switch) [19]. Like its family member BAG1, BAG3 can bind via its C-terminal conserved BAG domain to the ATPase domain of HSP70 proteins, thereby competing with BAG1 for HSP70 binding. Under physiological conditions, the BAG1-HSP70 chaperone complex directs poly-ubiquitinated proteins to the proteasome [20,21,22]. Under pathophysiological conditions, additionally BAG3 in concert with HSP70 and HSPB8 targets misfolded and aggregation-prone proteins to the autophagic-lysosomal degradation pathway. Therefore, the observed BAG1-BAG3 expression switch is also followed by a functional switch from proteasomal to autophagic degradation [19,23,24,25]. The BAG3-promoted autophagic degradation of substrates requires their previous retrograde transport along microtubules to the microtubule organization center (MTOC), followed by their sequestration in the perinuclearly located aggresome [24,26]. In detail, BAG3 forms a multiprotein complex together with the molecular chaperones HSP70 (HSPA8) and HSPB8 (and also other small HSPs like HSPB6, HSPB5, or HSPB1), the ubiquitin ligase STUB1/CHIP as well as the HSP40 protein family member DNAJB6 that recognizes substrates destined for degradation [19,23,24,27,28,29,30,31]. In concert with the 14-3-3γ protein, BAG3 associates this complex with the cytoplasmic dynein motor complex, thereby enabling the aggresomal targeting of the substrate [24,32]. Concomitantly, BAG3 mediates the induction of the selective macroautophagic turnover of client proteins by simultaneously complexing with the macroautophagy receptor protein p62/SQSTM1, which can bind to the cargo as well as to the autophagosome membrane-associated protein LC3 [19,24,25,28]. This BAG3-triggered selective macroautophagic pathway, also referred to as *BAG3-mediated selective macroautophagy*, is evolutionarily highly conserved [24,28] and functions as a crucial cellular safeguarding system in response to cellular stress [16,17,33,34].

The HSP70 co-chaperone BAG3 is ubiquitously expressed at low levels; a highly constitutive expression of BAG3 was found in myocytes of cardiac and skeletal muscles as well as in cells of various cancer types [35,36,37,38,39,40]. The expression of BAG3 is inducible by different types of cellular stress, including oxidative stress or stress caused by heat, heavy metals, virus infection, or proteasome inhibition (e.g., by MG132) [19,25,41,42,43,44,45,46]. This stress-induced upregulation of BAG3 is often caused by HSF1 (*heat shock transcription factor 1*) that binds to heat shock-responsive elements within the *BAG3* promoter [45,47,48,49,50]. Especially under oxidative stress, NRF2 (*nuclear factor erythroid 2-related factor 2*) has been shown to enhance *BAG3* gene expression [33,51]. Besides HSF1 and NRF2, other transcription factors, such as NF-κB (*nuclear factor NF-kappa-B*), EGR-1 (*early growth response protein 1*) or AIbZIP (*androgen-regulated protein androgen-induced bZIP*), the oncogene WT1 (*Wilm’s tumor 1 protein*), or even BAG3 itself are able to transcriptionally activate BAG3 expression [52,53,54,55,56,57,58]. At the protein level, activity and function of BAG3 are reported to be modulated by post-translational modifications, e.g., by phosphorylation. Several studies have demonstrated that the phosphorylation status of BAG3 is decisive for its interaction with other proteins and thus for its intracellular function [16].

The 75 kDa BAG3 displays a multi-modular protein structure, enabling the interaction with a wide variety of proteins. In addition to its binding to HSP70, the C-terminal BAG domain is described to interact with the anti-apoptotic protein BCL-2 (*B-cell lymphoma 2*) and with HSF1 [13,49,59,60]. Furthermore, BAG3 encompasses a WW (tryptophan-tryptophan) domain at its N-terminus that mediates the interaction with proteins containing proline-rich repeats, such as PDZGEF2 (*guanine nucleotide exchange factor 2*), the adenovirus penton base protein, SYNPO2 (*synaptopodin-2*), LATS1/2 (*large tumor suppressor homolog 1/2*), AMOTL1/2 (*angiomotin-like protein 1/2*), or TSC1 (*tuberous sclerosis 1*) [30,61,62,63,64]. The two conserved IPV (isoleucine-proline-valine) motifs at the N-terminus and in the middle of BAG3 serve as docking sites for the small heat shock proteins HSPB8 as well as HSPB6 (possibly also for HSPB5 and HSPB1) [23,65,66]. Via its PxxP (proline-rich) region, BAG3 can associate with the motor protein dynein and with SH3 (*SRC homology 3*) domains of proteins, as observed in PLC-γ (*phospholipase C gamma*) or the tyrosine kinase SRC [24,67,68]. The adaptor protein 14-3-3γ has been demonstrated to phosphorylation-dependently bind to two phosphoserine-containing motifs (RSXpS) of BAG3 [32]. In addition, studies revealed unmapped protein-protein interactions of BAG3 with proteins such as IFITM-2 (*interferon-induced transmembrane protein 2*) or STK38 (*serine/threonine-protein kinase 38*) [69,70,71,72]. Proteomic studies have fundamentally expanded the interactome of BAG3 [33,72,73,74,75,76,77].

As its diverse spectrum of interactors already indicates, BAG3 is implicated in the regulation of various cellular signaling pathways and basic biological processes [15,18,36,40]. Thus, BAG3 may act as a scaffolding protein that complexes proteins (e.g., HSP70) and direct them to their site of action. As an adaptive response to proteostasis stress, BAG3 has been demonstrated to bidirectionally interfere with the HSF1 pathway, the NRF2-KEAP1 signaling pathway, the Hippo pathway (e.g., LATS 1/2 or AMOTL 1/2) as well as the JNK (*jun N-terminal kinase*) and p38 MAPK (*mitogen-activated protein kinase*) pathways [30,31,33,49,51,72]. Besides its pivotal role in autophagy, the function of BAG3 in apoptosis ranks among its most important physiological tasks [42,78,79]. Moreover, the co-chaperone BAG3 is involved in the modulation of key cellular processes, including development, cytoskeletal organization and dynamics, cell cycle progression, or cellular metabolism [17,36,80,81]. Due to its relevance in the regulation of key pathways, the dysfunction or deregulation of BAG3, as observed in cancer, myopathies, and age-related neurodegenerative disorders, has a devastating effect on cells and tissues [16,36,40,82,83,84,85,86]. In cancer, BAG3 has been reported to be responsible for modulating processes such as cell survival, cell adhesion, metastasis and angiogenesis [36,40,86,87,88]. Notably, BAG3-mediated selective macroautophagy is crucial for the disposal of aggregated proteins associated with age-related neurodegenerative disorders, including Alzheimer’s disease (tau-protein), Huntington’s disease (mutant huntingtin/polyQ proteins), and amyotrophic lateral sclerosis (mutant SOD1) [24,27,51,89,90,91,92,93,94,95].

In cellular protein quality control, the BAG3-HSP70-HSPB8 complex represents a node for proteotoxicity-induced signaling to guarantee proteostasis [16,17,33,34]. To further clarify the extraordinary role of BAG3 and its macroautophagic pathway in cellular homeostasis, we performed BAG3 interaction profiling under basal and proteostasis stress conditions, defined specific BAG3 interactomes under these conditions and uncovered proteins whose protein-protein interaction with BAG3 was significantly altered in response to stress. To induce cellular proteostasis stress, we used a commonly employed and proven method in proteostasis research and blocked the proteasomal degradation pathway by the reversible proteasome inhibitor MG132 [96,97]. By functional annotation/enrichment analysis as well as protein-protein interaction (PPI) enrichment analysis of the identified BAG3 binding proteins, we revealed and confirmed the multi-faceted nature of BAG3 and its involvement in a variety of diverse key cellular processes and signaling pathways. Moreover, we identified several novel potential BAG3 interaction partners and verified the tyrosine kinase YES1 as a novel BAG3 interactor. Hence, this study clearly demonstrates the central position of BAG3 in the cellular proteostasis network.

## 2. Materials and Methods

### 2.1. Reagents

Unless stated otherwise, all reagents were obtained from regular commercial sources as mentioned in the methods descriptions. The following antibodies were used: anti-BAG3 (Proteintech Group, 10599-1-AP), anti-FLAG (Sigma-Aldrich, F1804, St. Louis, MO, USA), anti-HSP72 (Enzo Life Sciences, Farmingdale, NY, USA, ADI-SPA-810), HRP anti-mouse IgG (H+L) (Jackson ImmunoResearch Laboratories, West Grove, PA, USA, 715-035-151), Cy3 anti-mouse IgG (H+L) (Jackson ImmunoResearch Laboratories, 715-165-151), HRP anti-rabbit IgG (H+L) (Jackson ImmunoResearch Laboratories, 711-035-152), Alexa Fluor 647 anti-rabbit IgG (H+L) (Jackson ImmunoResearch Laboratories, 711-605-152), anti-rabbit IgG (Sigma-Aldrich, I5006), anti-tubulin (Sigma-Aldrich, T9026), and anti-YES1 (Sigma-Aldrich, HPA026480).

### 2.2. Cell Culture and Treatment of Cells

HEK293T cells were maintained in Dulbecco’s modified eagle medium (DMEM, Thermo Fisher Scientific, Waltham, MA, USA) supplemented with 1 mM sodium pyruvate (Thermo Fisher Scientific), 10% (*v*/*v*) fetal calf serum (FCS, Thermo Fisher Scientific) and a mixture of antibiotics and antimycotics (Thermo Fisher Scientific) at 37 °C in a 5% CO_2_-humidified atmosphere. The medium was refreshed every three days and 24 h prior to experiments. For proteasome inhibition, cells were treated with 10 µM MG132 (Calbiochem) (dissolved in DMSO (Roth)) for 6 h (48 h after seeding). DMSO (Roth) treatment (0.1%) was used as control (basal condition).

### 2.3. Plasmids and Transfection Method

To overexpress human BAG3 and a FLAG tagged version of human BAG3, the expression plasmids pBAG3-N1 and pFLAG-BAG3-N1 were used [19,24]. Expression plasmid for human FLAG tagged YES1 (pFLAG-hYES1) was constructed by cloning partial human YES1 cDNA containing the whole CDS into pN1. The forward primer sequence used to clone YES1 plasmid is 5′-TTT GAT CAG GCT GCA TTA AAA GTA AAG AAA-3′ and the reverse primer sequence is 5′-TTT CTA GAT TTT ATA AAT TTT CTC CTG GCT-3′. After vector linearization with BamHI and XbaI, PCR products were inserted using In-Fusion cloning (Clontech) according to the manufacturer’s protocol. The sequence of this construct was verified by sequencing analysis (MWG).

For exogenous overexpression of proteins, cells were transfected by calcium phosphate precipitation. In brief, plasmids were added to pre-warmed (60 °C) calcium-solution [250 mM CaCl_2_ in DEPC-H_2_O (Thermo Fisher Scientific)] and mixed with an equal amount of pre-warmed 2× phosphate-buffer (280 mM NaCl, 50 mM HEPES, 1.5 mM Na_2_HPO_4_, pH 7.1). Transfection-solution was incubated for 30 min at RT and added to the medium subsequently. The medium was refreshed after 16 h and cells were harvested 48 h after transfection.

### 2.4. Affinity Purification for qAP-MS

Cells were washed with ice-cold phosphate-buffered saline (PBS, Sigma-Aldrich), lysed in ice-cold lysis buffer (50 mM Tris-HCl (pH 7.4), 150 mM NaCl, 2 mM EDTA, 0.5 mM EGTA, 10% glycerol, 1% Triton X-100, phosphatase and EDTA-free protease inhibitor cocktails (Roche)) and incubated on ice for 15 min. After centrifugation of the crude cell extract at 1000× *g* for 10 min at 4 °C, the protein concentration of the supernatant was determined using the bicinchoninic acid protein assay (Thermo Fisher Scientific) according to the manufacturer’s protocol. Total protein concentration was adjusted to approximately 1 µg/µL with lysis buffer. To pre-clear the lysate of non-specifically binding proteins, 25 µL of washed and equilibrated Pierce Protein A/G Magnetic Beads (Thermo Fisher Scientific) were added to 500 µL protein lysate (~500 µg) in a low retention tube and incubated on a rotator at 4 °C for 1 h. 1% of the lysate (~5 µg) was used as input control. Following centrifugation at 1000× *g* at 4 °C for 15 min, 1–2 µg of BAG3 antibody (DMSO_IP and MG132_IP) or 1–2 µg of corresponding IgG antibody (IP control: MG132_IgG) were added to the cleared supernatant and incubated with agitation overnight at 4 °C (see Section 2.1). The next day, 25 µL of new, washed and equilibrated magnetic beads were added to the antibody-lysate suspension and incubated on a rotator at 4 °C for 1–2 h. Afterwards, the beads were washed (1000× *g*, 4 °C, 1 min) five times with 1 mL wash buffer (50 mM Tris-HCl (pH 7.4), 200 mM NaCl, 2 mM EDTA, 0.5 mM EGTA, 1% Triton X-100, phosphatase and EDTA-free protease inhibitor cocktails (Roche)). To elute BAG3 and co-immunoprecipitated proteins from beads, the bead pellet was resuspended in 35 µL of 2× loading buffer [4% sodium dodecyl sulfate, 8% glycerol, 50 mM Tris, 0.4 mM EDTA, 0.0008% bromophenol-blue, 4% β-mercaptoethanol] and incubated at 25 °C for 10 min with agitation. Finally, the sample was cleared (21,000× *g*, 4 °C, 1 min) and the supernatant was loaded on an SDS-PAGE gel.

### 2.5. In-Gel Digestion for Mass Spectrometry

For in-gel digestion, gel lanes were cut into pieces and digestion was performed as described previously [98]. In brief, gel pieces were washed, destained and dehydrated. Proteins were reduced with 10 mM dithiothreitol (DTT), alkylated with 55 mM iodoacetamide (IAA) and digested overnight with sequencing-grade Trypsin (Promega). Peptides were extracted using an increasing concentration of acetonitrile. Finally, peptides were concentrated and desalted using the stop and go extraction (STAGE) technique [99].

### 2.6. Liquid Chromatography and Mass Spectrometry

A binary buffer system consisting of buffer A (0.1% formic acid) and buffer B (80% acetonitrile, 0.1% formic acid) was used for peptide separation on an Easy-nLC 1200 (Thermo Fisher Scientific). This system was coupled via a nano electrospray ionization source to the quadrupole-based Q Exactive HF benchtop mass spectrometer [100]. Peptide elution from the in-house packed 18 cm (1.9 µm C18 Beads, Dr. Maisch GmbH) column was achieved by increasing the relative amount of B from 10% to 38% in a linear gradient within 23 min at a column temperature of 40 °C. Followed by an increase to 100% B within 7 min, gradients were completed by a re-equilibration to 5% B. Q Exactive HF settings: MS spectra were acquired using 3E6 as an AGC target, a maximal injection time of 20 ms and a 60,000 resolution at 200 m/z. The mass spectrometer operated in a data dependent Top15 mode with subsequent acquisition of higher-energy collisional dissociation (HCD) fragmentation MS/MS spectra of the top 15 most intense peaks. Resolution for MS/MS spectra was set to 30,000 at 200 m/z, AGC target to 1E5, maximal injection time to 64 ms and the isolation window to 1.6 Th.

### 2.7. Mass Spectrometry Data Processing and Analysis

All acquired raw files were processed using MaxQuant (version 1.5.3.30) and the implemented Andromeda search engine [101,102]. For protein assignment, electrospray ionization-tandem mass spectrometry (ESI-MS/MS) fragmentation spectra were correlated with the Uniprot human database (2017) including a list of common contaminants. Searches were performed with tryptic specifications and default settings for mass tolerances for MS and MS/MS spectra. Carbamidomethyl at cysteine residues was set as a fixed modification, while oxidation at methionine and acetylation at the N-terminus were defined as variable modifications. The minimal peptide length was set to seven amino acids and the false discovery rate for proteins and peptide-spectrum matches to 1%. The match-between-run feature was used with a time window of 0.7 min. Relative label-free quantification of proteins was done using the MaxLFQ algorithm integrated into MaxQuant [103]. The minimum LFQ ratio count was set to 2 and the FastLFQ option was enabled.

Perseus software (version 1.6.12.0) was used for further statistical analysis [104]. Data were filtered for contaminants, reverse entries and proteins that were only identified by site. Moreover, proteins identified by only one peptide or one unique peptide were removed (Relation: x > 1). The LFQ intensities were logarithmized (log2 scale) and grouped into triplicates. DMSO_IP1-DMSO_IP3 refer to the three independent biological replicates of the BAG3 IP samples generated from DMSO-treated cells, MG132_IP1-MG132_IP3 refer to the three independent biological replicates of the BAG3 IP samples generated from MG132-treated cells and MG132_IgG1-MG132_IgG3 refer to the three independent biological replicates of the IgG IP samples generated from MG132-treated cells. The respective three biological replicates were grouped to DMSO_IP, MG132_IP and MG132_IgG. Furthermore, three valid values were required in at least one group. Missing LFQ intensities (NaN) were imputed based on low values from a normal distribution (width ₌ 0.3; down shift ₌ 1.8) (for histogram, see Supplementary Figure S1). To evaluate the quality of the data set, a two-dimensional principal component analysis (PCA; number of components: 2, cutoff method: *p*-value, *p*-value threshold; 0.05), a multi scatter plot of the LFQ intensities of the corresponding samples with Pearson’s correlation coefficients as well as heat maps and hierarchical clustering (rows and columns tree: distance: euclidean, linkage: average, preprocess with K-means, number of clusters: 300, maximal number of iterations: 10, number of restarts: 1; Z-score: matrix access: rows; use median) were conducted. To compare two groups and determine significance, unpaired Student’s t-tests were performed (Setting: S0 ₌ 0; *p*-value: 0.05). STRING (Search Tool for the Retrieval of Interacting Genes/Proteins) database (version 11.0) was utilized for Gene Ontology (GO) functional annotation and enrichment analysis as well as for protein-protein interaction (PPI) enrichment and constructing of a PPI network of identified proteins (basic settings: meaning of network edges: confidence; active interaction sources: text mining, experiments, databases, co-expression, neighborhood, gene fusion, co-occurrence; minimum required interaction score: medium confidence (0.400); statistical background: whole genome).

### 2.8. Co-Immunoprecipitation

Cells were washed with ice-cold PBS (Sigma-Aldrich), lysed in ice-cold lysis buffer [50 mM Tris-HCl (pH 7.4), 150 mM NaCl, 2 mM EDTA, 0.5 mM EGTA, 10% glycerol, 1% Triton X-100, phosphatase and EDTA-free protease inhibitor cocktails (Roche)] and incubated on ice for 15 min. After centrifugation of the crude cell extract at 1000× *g* for 10 min at 4 °C, protein concentration of the supernatant was determined using the bicinchoninic acid protein assay (Thermo Fisher Scientific) according to the manufacturer’s protocol. Total protein concentration was adjusted to approximately 1 µg/µL with lysis buffer. 1% of the lysate (~5 µg) was used as input control. 25 µL of washed and equilibrated EZview Red ANTI-FLAG M2 Affinity Gel (Sigma Aldrich) were added to 500 µL protein lysate (~500 µg) in a low retention tube and incubated with agitation overnight at 4 °C. The next day, the beads were washed (1000× *g*, 4 °C, 1 min) five times with 1 mL wash buffer (50 mM Tris-HCl (pH 7.4), 150 mM NaCl, 2 mM EDTA, 0.5 mM EGTA, 1% Triton X-100, phosphatase, and EDTA-free protease inhibitor cocktails (Roche)). For elution, the bead pellet was resuspended in 35 µl of 2× loading buffer (4% sodium dodecyl sulfate, 8% glycerol, 50 mM Tris, 0.4 mM EDTA, 0.0008% bromophenol-blue, 4% β-mercaptoethanol) and incubated at 99 °C for 5 min with agitation. Finally, the sample was cleared (21,000× *g*, 4 °C, 1 min) and the supernatant as well as the input sample were loaded on SDS-PAGE gels.

### 2.9. Immunoblotting

Samples were boiled for 5 min at 99 °C, subjected to denaturing SDS-PAGE and subsequently transferred to nitrocellulose membranes. Membranes were incubated with PBS containing 0.05% Tween20 (PBST) and 4% non-fat dried milk powder (Applichem) for 1 h at RT to block unspecific binding sites. All antibodies were dissolved in PBST for incubation (see Section 2.1). Primary antibodies were detected by chemiluminescence using peroxidase-conjugated secondary antibodies (see Section 2.1 Reagents) and enhanced chemiluminescence-developing buffer (0.025 % luminol (Sigma-Aldrich), 0.1 M Tris-HCl pH 8.6, 0.011% p-coumaric acid (Sigma-Aldrich), 0.3% H_2_O_2_). Chemiluminescent signals were detected with the Amersham Imager 600 system (GE Scientific).

### 2.10. Immunocytochemistry

Glass cover slips used for HEK293T cells were coated with 10 µg/mL poly-L-ornithine (Sigma-Aldrich) for 1 h at 37 °C and subsequently washed twice with PBS (Sigma-Aldrich) and once with culture medium. HEK293T cells were grown on glass cover slips and fixed with ice-cold 4% paraformaldehyde containing buffer (Rotifix; Roth) for 15 min at RT. Permeabilization of cells and blocking of unspecific binding sites were performed with PBS containing 0.1% Triton X-100 (Sigma-Aldrich) and 3% bovine serum albumin (BSA; Sigma-Aldrich) for 5 min at 4 °C. Cells were incubated with primary antibodies (see Section 2.1 Reagents) diluted in PBS containing 1% BSA at 4 °C overnight. Thereafter, cells were incubated with fluorophore-conjugated secondary antibodies (see Section 2.1 Reagents) diluted in PBS containing 1% BSA for 1 h at RT. Laser scanning confocal microscopy was performed using the Zeiss LSM 710 system.

## 3. Results

### 3.1. Establishment of BAG3-Proteostasis-Stress-Interactome

**Figure 1:**
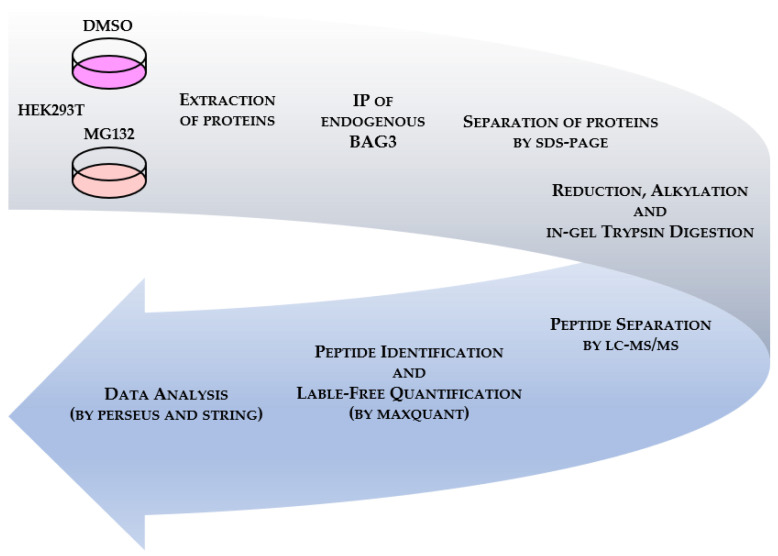
Methodological workflow to establish BAG3 interactomes under basal and proteostasis stress conditions via affinity purification combined with quantitative mass spectrometry (qAP-MS). Briefly, HEK293T cells were treated either with DMSO or 10 µM MG132 for 6 h. After extraction of proteins, endogenous BAG3 was immunoprecipitated and the eluates were separated by SDS-PAGE. Following reduction, alkylation and in-gel trypsin digestion, peptides were analyzed by LC-MS/MS. Raw files were processed using MaxQuant. Relative label-free quantification (LFQ) was performed with MaxLFQ algorithm integrated into MaxQuant. Statistical data analysis was conducted by Perseus software followed by functional annotation and PPI enrichment analysis via STRING database.

To identify new interaction partners of BAG3 and proteins whose interaction with BAG3 are modulated under proteostasis stress conditions, we established BAG3 interactomes under basal conditions and upon proteasome inhibition (Figure 1). To map the altered BAG3 interactome upon proteasome failure, HEK293T cells were treated with the reversible proteasome inhibitor MG132 for 6 h. DMSO-treated HEK293T cells were used as control (basal conditions). Blocking the proteasomal degradation pathway by MG132 leads to the accumulation and aggregation of (poly-) ubiquitinated proteins, thereby inducing the BAG3-mediated aggresome formation and promoting selective macroautophagy (BAG1-BAG3-switch/proteasome-to-autophagy-switch) [19,25,105]. In addition, MG132 has been shown to increase the expression of BAG3 at the transcriptional level in a HSF1-dependent manner, thereby enhancing the BAG3-mediated selective macroautophagy pathway, too [44,45].

To define BAG3 protein-protein interactions (PPIs) under the described conditions, we employed quantitative affinity purification coupled to mass spectrometry (qAP-MS) (Figure 1). After extraction of total proteins, endogenous BAG3 was immunoprecipitated and the IP eluates were separated by SDS-PAGE (the quality and specificity of the performed BAG3 IP was validated by analyzing aliquots of input and IP samples via immunoblotting (Supplementary Figure S2A,B)). Following reduction, alkylation and in-gel trypsin digestion of the proteins, peptides were separated by nano liquid chromatography high-resolution tandem mass spectrometry (nLC-MS/MS). Raw data were processed using MaxQuant software with the build-in search engine Andromeda, identifying proteins via sequences provided by Uniprot human database. Relative label-free quantification (LFQ) was conducted by the MaxLFQ algorithm integrated into MaxQuant. The MaxQuant output data were statistically analyzed by Perseus software; STRING (Search Tool for the Retrieval of Interacting Genes/Proteins) database was utilized to elucidate the functional annotation/enrichment and the PPI network of identified BAG3 binding proteins. Cell treatment and an immunoprecipitation assay were performed in three independent biological replicates.

### 3.2. BAG3 Interaction Profiling under Basal and Proteostasis Stress Conditions

**Figure 2:**
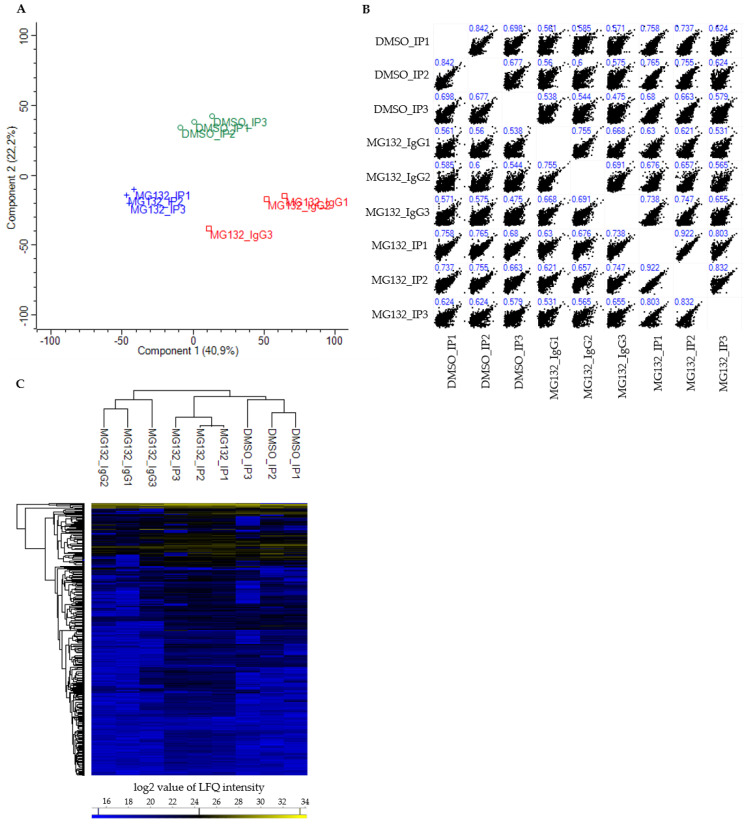
Multivariate statistical analysis of processed BAG3 interactome data set by Perseus software. (**A**) Principal component analysis (PCA) of LFQ intensities of all proteins identified in the respective samples. Replicates of DMSO_IP (DMSO_IP1-DMSO_IP3) are shown as green open circles, replicates of MG132_IP (MG132_IP1-MG132_IP3) are shown as blue crosses and replicates of the IP control MG132_IgG (MG132_IgG1-MG132_IgG3) are shown as red open squares (Number of components: 2; Cutoff method: *p*-value = 0.05). (**B**) Multi scatter plot of LFQ intensities of all proteins identified in DMSO_IP, MG132_IP and MG132_IgG with Pearson’s correlation coefficients (blue numbers). (**C**) Hierarchical clustering and heat map of LFQ intensities of proteins identified in DMSO_IP, MG132_IP and MG132_IgG. The three groups DMSO_IP, MG132_IP and MG132_IgG including their three biological replicates are clustered in columns and proteins are clustered in rows. Color scale reports log2-transformed LFQ intensity values; blue indicates a high LFQ intensity, yellow marks a low LFQ intensity.

Processing of the proteomic interaction data set by Perseus resulted in the identification of 1335 proteins in total (Supplementary Table S1). To evaluate the quality of the generated BAG3 interactome data set, we performed a two-dimensional principal component analysis (PCA) as well as a hierarchical clustering, and created a multi scatter plot of the LFQ intensities of all proteins detected in the corresponding samples (Figure 2).

The PCA score plot showed that the DMSO_IP replicates (DMSO_IP1-DMSO_IP3) as well as the MG132_IP replicates (MG132_IP1-MG132_IP3) clustered tightly together, the replicates of the IP control MG132_IgG (MG132_IgG1-MG132_IgG3) formed a third more loose cluster (Figure 2A). Nevertheless, all three analyzed groups (DMSO_IP, MG132_IP and MG132_IgG) were clearly separated from each other with a variance of component 1 of 40.9% and a variance of component 2 of 22.2% (Figure 2A). The multi scatter plot of the respective LFQ intensities and the corresponding Pearson’s correlation coefficients illustrated a moderate to strong correlation between the three different groups (DMSO_IP, MG132_IP and MG132_IgG) and within the replicates of one group (Figure 2B). To visualize the protein abundance in all replicates, we carried out a hierarchical clustering and a heat map of the LFQ intensities of all detected proteins (Figure 2C). As already revealed by PCA, the three independent biological replicates of each group clustered strongly together. Here, the replicates of DMSO_IP and MG132_IP correlated more than the replicates of DMSO_IP and MG132_IgG or the replicates of MG132_IP and MG132_IgG. A majority of the identified proteins was detected in all three groups equally and at low abundance. However, the heat map additionally revealed that the LFQ intensities of a portion of proteins were altered in DMSO_IP and MG132_IP compared to the IP control MG132_IgG and also differed in DMSO_IP and MG132_IP (Figure 2C). Taken together, these data point to enriched interaction partners of BAG3 in DMSO_IP and MG132_IP in comparison to control MG132_IgG and a changed quantitative binding of specific proteins to BAG3 upon proteasome inhibition with MG132.

**Figure 3:**
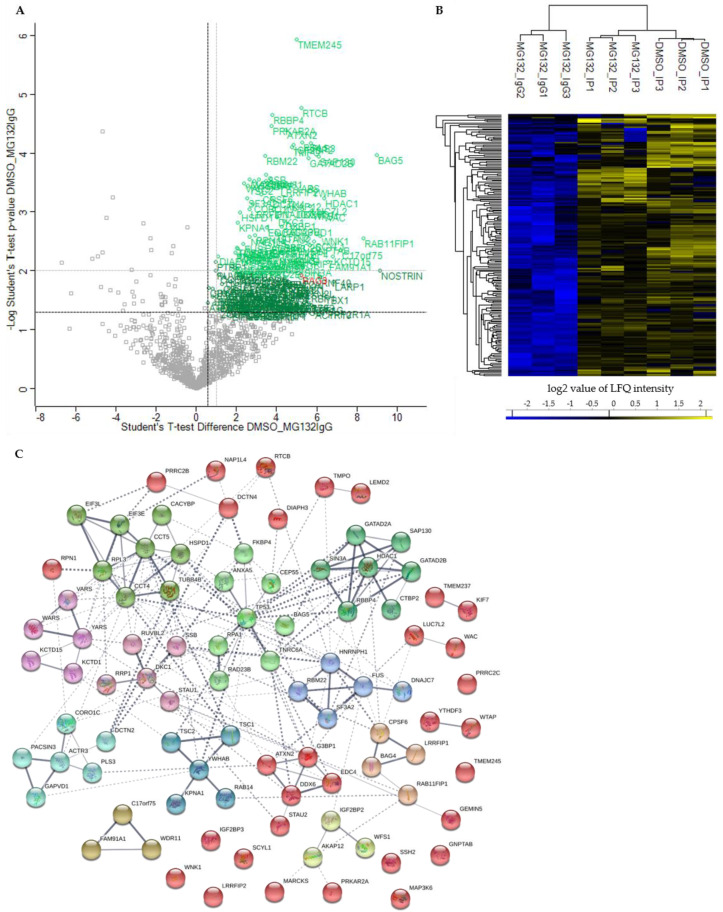
Quantitative proteomic analysis of the BAG3 interactome under basal conditions (DSMO_IP). (**A**) Scatter plot generated by plotting the log2 ratios against the negative log10 *p*-values of the Student’s t-test DMSO_IP compared to MG132_IgG (DMSO_MG132IgG; *p*-value = 0.05; S0 = 0). Proteins with a *p*-value ≤ 0.05 and a ratio ≥ 1.5 (black dash lines) were considered as significant, labelled with the gene symbols of the respective proteins and are shown as green open circles. BAG3 as bait protein was marked with red. Class A BAG3 interactors with a *p*-value ≤ 0.01 and a ratio ≥ 2 (grey dash lines) are depicted as light green open circles. (**B**) Hierarchical clustering and heat map of LFQ intensities of all significant BAG3 interactors (*p*-value ≤ 0.05 and ratio ≥ 1.5) under basal conditions (DMSO_IP). The three groups DMSO_IP, MG132_IP and MG132_IgG including their three biological replicates are clustered in columns and proteins are clustered in rows. Color scale reports Z-scored log2-transformed LFQ intensity values; blue indicates a high LFQ intensity, yellow marks a low LFQ intensity. (**C**) Protein-protein interaction (PPI) network of class A BAG3 interactors (*p*-value ≤ 0.01 and ratio ≥ 2) under basal conditions (DMSO_IP). Network was clustered into 12 subnetworks using the k-means clustering method; nodes/proteins of the same cluster exhibit the same color. Thickness of connecting lines/edges correlates with the strength of the association. Further network parameters: number of nodes: 90, number of edges: 203 (expected: 89), average node degree: 4.51, avg. local clustering coefficient: 0.46, PPI enrichment *p*-value < 1.0e-16, statistical background: whole genome. Scatter plot including Student’s t-test and hierarchical clustering/heat map were performed by Perseus software and the PPI network was created by the STRING database.

**Figure 4:**
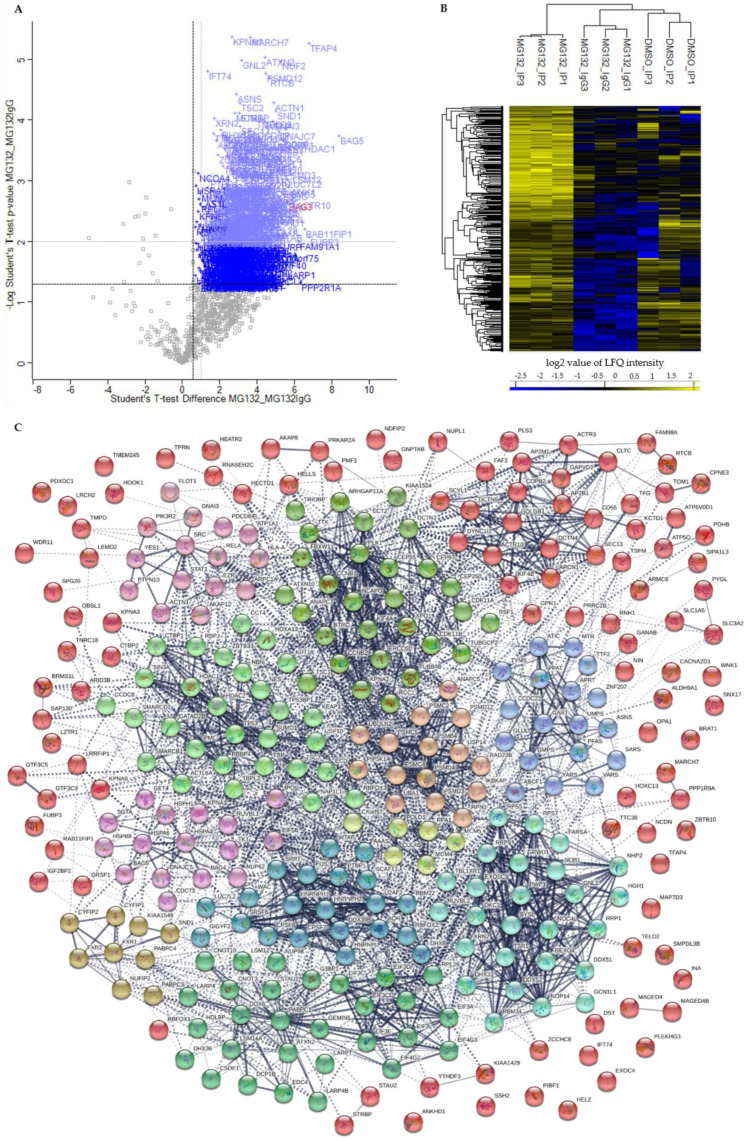
Quantitative proteomic analysis of the BAG3 interactome upon proteasome inhibition (MG132_IP). (**A**) Scatter plot generated by plotting the log2 ratios against the negative log10 *p*-values of the Student’s t-test MG132_IP compared to MG132_IgG (MG132_MG132IgG; *p*-value = 0.05; S0 = 0). Proteins with a *p*-value ≤ 0.05 and a ratio ≥ 1.5 (black dash lines) were considered as significant, labelled with the gene symbols of the respective proteins and are shown as blue crosses. BAG3 as bait protein was marked with red. Class A BAG3 interactors with a *p*-value ≤ 0.01 and a ratio ≥ 2 (grey dash lines) are depicted as light blue crosses. (**B**) Hierarchical clustering and heat map of LFQ intensities of all significant BAG3 interactors (*p*-value ≤ 0.05 and a ratio ≥ 1.5) upon proteasome inhibition (MG132_IP). The three groups DMSO_IP, MG132_IP and MG132_IgG including their three biological replicates are clustered in columns and proteins are clustered in rows. Color scale reports Z-scored log2-transformed LFQ intensity values; blue indicates a high LFQ intensity, yellow marks a low LFQ intensity. (**C**) Protein-protein interaction (PPI) network of class A BAG3 interactors (*p*-value ≤ 0.01 and ratio ≥ 2) upon proteasome inhibition (MG132_IP). Network was clustered into 12 subnetworks using the k-means clustering method; nodes/proteins of the same cluster exhibit the same color. Thickness of connecting lines/edges correlates with the strength of the association. Further network parameters: number of nodes: 319, number of edges: 2160 (expected: 1216), average node degree: 13.5, avg. local clustering coefficient: 0.429, PPI enrichment *p*-value < 1.0e-16, statistical background: whole genome. Scatter plot including Student’s t-test and hierarchical clustering/heat map were performed by Perseus software and the PPI network was created by the STRING database.

To distinguish between specific interactors of BAG3 and background binders, a statistical analysis of the LFQ intensities of proteins detected in DMSO_IP and MG132_IP compared to their LFQ intensities detected in the IP control MG132_IgG was conducted by a two-sample analysis (Student’s t-test, *p*-value = 0.05, S0 = 0). First, identified proteins with a *p*-value ≤ 0.05 and a ratio ≥ 1.5 were considered as significant. In comparison to control MG132_IgG, we could identify in total 191 significant potential BAG3 interactors under basal conditions (DMSO_IP) and the immense quantity of 561 significant potential BAG3 interactors upon proteasome inhibition (MG132_IP) (Figure 3A, green labelled proteins; Figure 4A, blue labelled proteins; Supplementary Tables S2 and S3). In both cases, BAG3 was found among the significantly enriched binders (Figure 3A and Figure 4A, red labelled), indicating for the specificity and a good quality of the performed BAG3 immunoprecipitation. The higher protein abundance of the identified specific BAG3 interactors in DMSO_IP and MG132_IP compared to MG132_IgG was additionally visualized by heat maps and hierarchical clustering (Figure 3B and Figure 4B). To cut down the list of significant interactors and to determine the top 25 significantly enriched BAG3 interactors under basal and proteostasis stress conditions, we strengthened the significance criteria and filtered for proteins with a *p*-value ≤ 0.01 and a ratio ≥ 2 (class A interactors). Thus, 91 proteins were defined as class A BAG3 interactors under basal conditions (DMSO_IP) and 309 proteins were defined as class A BAG3 interactors under proteostasis stress conditions (MG132_IP) (Figure 3A, light green labelled proteins; Figure 4A, light blue labelled proteins; Supplementary Tables S4 and S5).

Among the top 25 enriched class A BAG3 interactors detected under basal conditions (DMSO_IP), the BAG protein family member BAG5, the transmembrane protein TMEM245, the RNA-binding protein LUC7L2, the adaptor proteins YWHAB, the enzyme GlcNAc-1-phosphotransferase GNPTAB, the two components of the Sin3/HDAC complex HDAC1 and SIN3A, the Sin3/HDAC complex-associated protein SAP130, the tRNA-splicing ligase subunit RTCB, the cytoskeleton-linked proteins KIF7 and PLS3 as well as transcriptional repressor proteins such as KCTD1, KCTD15, WAC and GATAD2B were found (Table 1). The enriched proteins RAB11FIP1, SCYL1 and the components of the WDR11 complex NJMU-R1 and FAM91A1 are supposed to be involved in cellular transport processes. The listed proteins WNK1 and WFS1 are both described to be implicated in regulating electrolyte homeostasis. Moreover, the TriC (*T-complex protein ring complex*) complex subunit CCT5, the RNA helicase DDX6, the isomerase FKBP4 and RPA1, a part of the replication protein A complex, were detected to be highly enriched potential BAG3 interactors under basal conditions (Table 1). Interestingly, BAG5, HDAC1, RAB11FIP1, LUC7L2, DDX6, PLS3, WNK1, RPA1 and SCYL1 also belong to the top 25 hits of enriched class A BAG3 interactors identified under proteostasis stress conditions (MG132_IP) (Table 2). Beside the DNAJ/HSP40 family member DNAJC7 and the ribosome subunit RPS7, the protein LSM12, the endonuclease SND1, the glutamine synthetase GLUL, the helicase G3PB1, the transcription regulating proteins TFAP4 and FUBP3 as well as the cytoskeleton-associated proteins ACTR10 and ACTN1 represented significantly enriched potential BAG3 interactors detected in MG132_IP (Table 2). Additionally, the components EIF3L and EIF3C of the eIF-3 (*eukaryotic translation initiation factor*) complex, the subunit Nuf2 of the kinetochore-essential NDC80 complex, the subunit GNAI3 of the G protein (*guanine nucleotide-binding protein*), as well as the subunits PMSC5 and PMSD3 of the 26S proteasome complex, were among the top 25 hits of potential BAG3 interactors found upon proteasome inhibition (MG132_IP) (Table 2).

To get more global insights into the cellular function and localization of the class A BAG3 interactors (*p*-value ≤ 0.01 and ratio ≥ 2) identified under basal conditions and upon proteasome inhibition, a list of these proteins was subjected to the STRING database for Gene Ontology (GO) functional annotation and enrichment analysis as well as for creating PPI networks (DMSO_IP: 90 proteins; MG132_IP: 319 proteins; Supplementary Tables S4 and S5). Beside the category Biological Process, the categories Molecular Function and Cellular Component were analyzed in detail.

**Figure 5:**
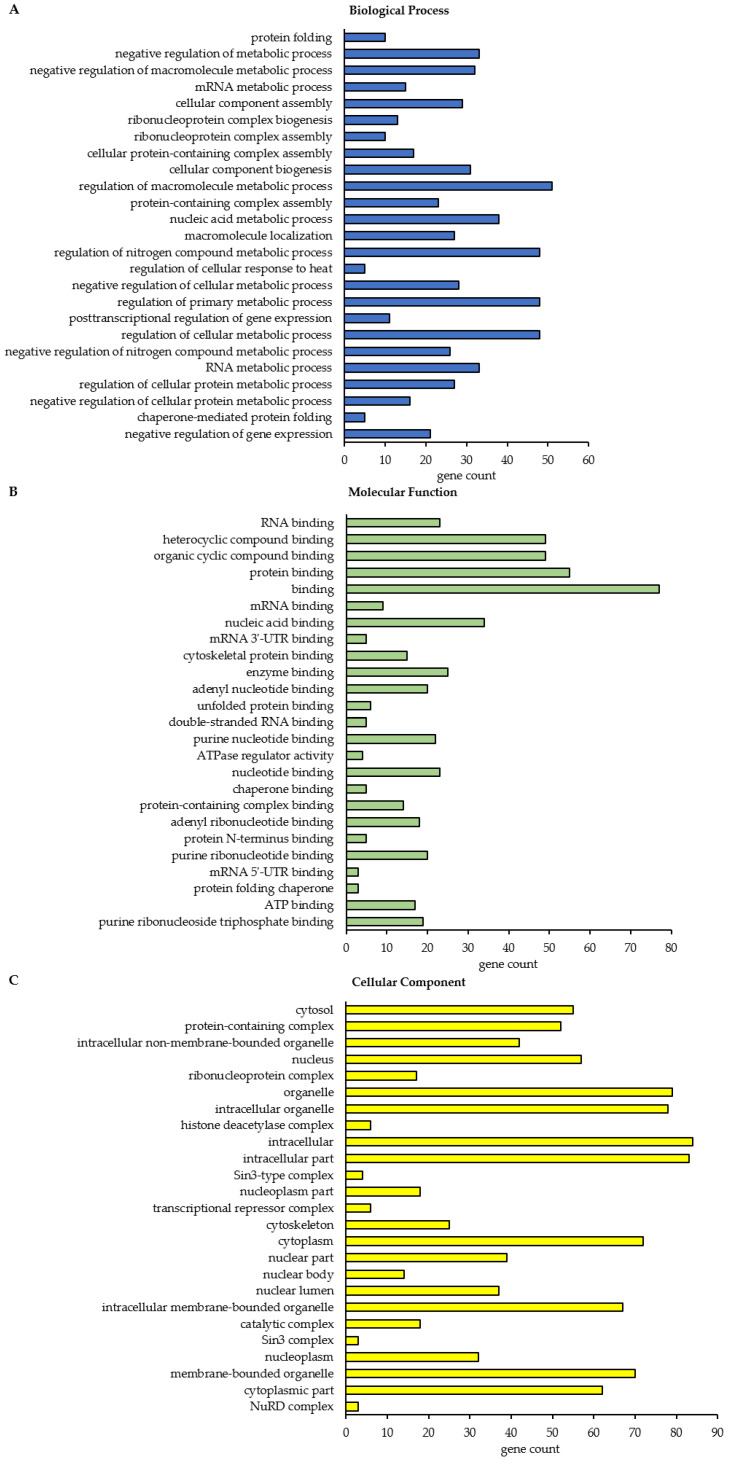
Gene ontology (GO) functional annotation and enrichment analysis of the BAG3 interactome under basal conditions (DMSO_IP). Only class A BAG3 interactors with a *p*-value ≤ 0.01 and a ratio ≥ 2 were subjected to analysis. The 25 most significantly enriched GO terms in the category Biological Process (**A**), Molecular Function (**B**) and Cellular Component (**C**) are shown. For each GO term, the observed gene count is presented. Analysis was performed by STRING database.

**Figure 6:**
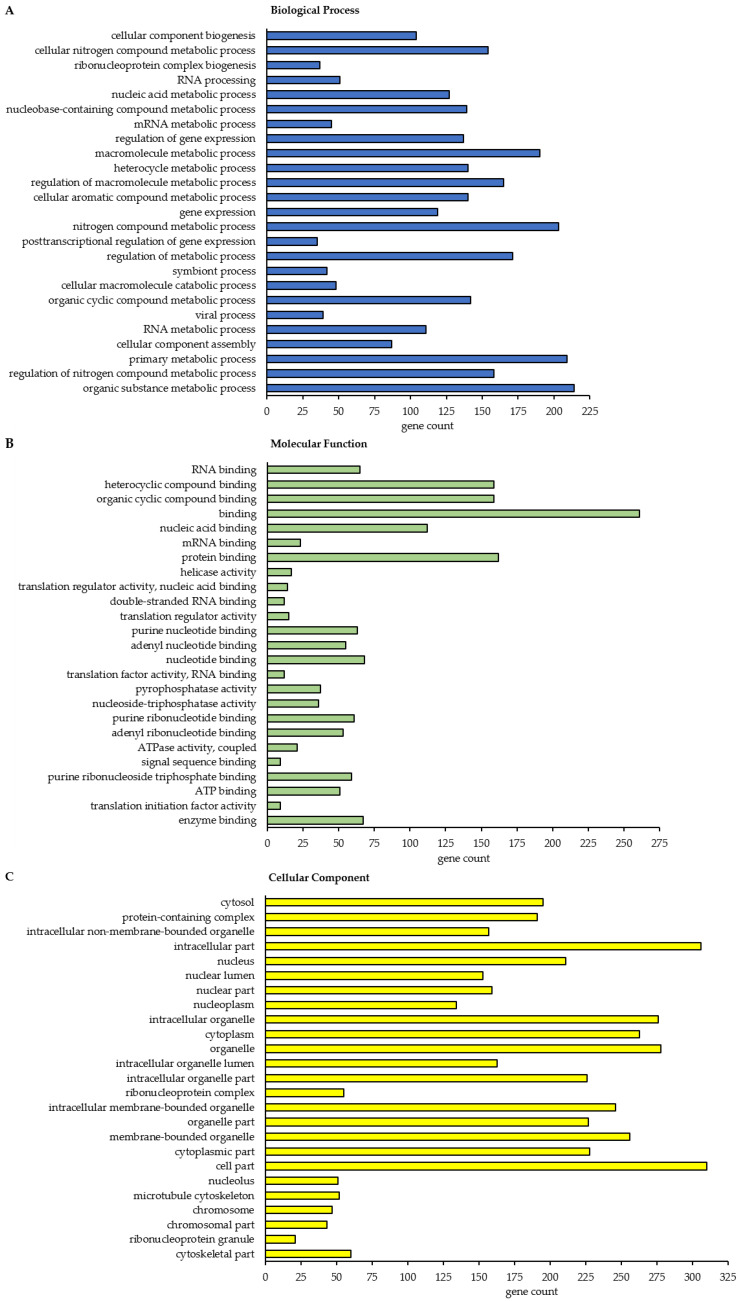
Gene ontology (GO) functional annotation and enrichment analysis of the BAG3 interactome upon proteasome inhibition (MG132_IP). Only class A BAG3 interactors with a *p*-value ≤ 0.01 and a ratio ≥ 2 were subjected to analysis. The 25 most significantly enriched GO terms in the category Biological Process (**A**), Molecular Function (**B**) and Cellular Component (**C**) are shown. For each GO term, the observed gene count is presented. Analysis was performed by STRING database.

In total, this STRING analysis revealed 114 different enriched GO terms related to Biological Process for class A BAG3 interactors detected under basal conditions and 404 distinct enriched GO terms for class A BAG3 interactors detected upon proteasome inhibition by MG132 (Supplementary Tables S6 and S7). Among the top 25 most enriched FDR-ranked Biological Process GO terms that were determined for potential BAG3 interactors detected under basal conditions, we found various GO terms linked to metabolic processes and their regulation, in most cases it was a negative regulation (Figure 5A; Table A1). In particular, RNA/mRNA metabolic processes, the regulation of gene expression as well as the cellular process protein folding were numbered among these 25 top hits of GO terms. GO terms associated with cellular component biogenesis, including cellular component or protein-containing complex assembly (especially of ribonucleoprotein complexes), and with macromolecule localization were also functionally annotated for the potential BAG3 interactors identified in DMSO_IP. Moreover, the GO term regulation of cellular response to heat stress was significantly enriched among the determined top 25 Biological Process GO terms under basal conditions. As for BAG3 binding proteins detected under basal conditions, GO terms related to component/complex biogenesis and assembly were high FDR-ranked among the top 25 enriched Biological Process GO terms that were defined for BAG3 interactors detected under proteostasis stress conditions (MG132_IP) (Figure 6A; Table A2). Various metabolic processes, including RNA metabolic processes (e.g., RNA processing) and gene expression, were also found among the 25 top hits of GO terms that were determined for BAG3 interactors identified in MG132_IP. Additionally, catabolic macromolecule processes were highly significantly enriched among the determined top 25 Biological Process GO terms upon proteasome inhibition. In contrast to the STRING analysis of the BAG3 interactors identified in DMSO_IP, the corresponding functional enrichment analysis of the BAG3 interactors detected in MG132_IP assigned the GO term symbiont process and in detail the GO term viral process to the 25 top hits of Biological Process GO terms. Regarding the category Molecular Function, we identified 49 enriched GO terms for the potential BAG3 interactors detected under basal conditions (DMSO_IP) and 111 enriched GO terms for the potential BAG3 interactors detected under proteostasis stress conditions (MG132_IP) (Supplementary Tables S8 and S9). In both cases, the list of the top 25 FDR-ranked enriched Molecular Function GO terms mostly contained GO terms linked to binding functions (Figure 5B and Figure 6B; Table A3 and Table A4). Beside nucleic acid binding (mainly RNA/mRNA-binding) and nucleotide binding, protein binding, including enzyme binding, unfolded protein binding, cytoskeletal protein binding and protein-containing complex binding, was significantly enriched, especially in the top 25 list of GO terms found for class A BAG3 interactors under basal conditions. While the functional enrichment analysis ranked activities such as ATPase regulator activity and protein folding chaperone among the top 25 enriched Molecular function GO terms that were defined for BAG3 interactors identified in DMSO_IP, GO terms concerning activities such as helicase activity, ATPase activity, phosphatase activity, translation regulator activity, translation factor activity or translation initiation factor activity were allocated to the top 25 hits of GO terms that were found for BAG3 interactors detected in MG132_IP (Figure 6B; Table A4). In the category Cellular Component, the STRING analysis resulted in the identification of 58 enriched GO terms for the class A BAG3 interactors detected under basal conditions and 118 enriched GO terms for the class A BAG3 interactors detected upon proteasome inhibition with MG132 (Supplementary Tables S10 and S11). Noteworthy, almost all identified class A BAG3 interactors under basal and proteostasis stress conditions were functionally annotated as intracellular/intracellular part. Under both conditions, Cellular Component GO terms associated with nuclear components (e.g., nucleus, nuclear part, nuclear lumen, nucleoplasm or nucleolus) and cytoplasmic components were the GO terms with the highest significance (Figure 5C and Figure 6C; Table A5 and Table A6).

Moreover, the GO term protein-containing complex, in particular ribonucleoprotein complex, was revealed as highly enriched. Protein-containing complexes such as histone deacetylase complexes (Sin3-type complex) or transcriptional repressor complexes (NuRD complex) were dedicated to the top 25 list of Cellular Component GO terms that were determined for BAG3 interactors identified in DMSO_IP. Both top 25 hit lists of Cellular Component GO terms additionally comprised GO terms grouped under the term organelle (membrane bounded or non-membrane bounded). Cytoskeleton, chromosome as well as ribonucleoprotein granule have to be highlighted as significantly enriched non-membrane bounded organelles in the top 25 enriched Cellular Component GO terms that were defined for BAG3 interactors detected in MG132_IP.

The PPI networks generated by the STRING database encompassed 90 nodes (equivalent to proteins) and 203 edges (equivalent to interactions) for the BAG3 interactome (class A interactors; *p*-value ≤ 0.01 and ratio ≥ 2) under basal conditions (DMSO_IP) and 319 nodes and 2160 edges for the BAG3 interactome upon proteasome inhibition (MG132_IP) (Figure 3C and Figure 4C). The enrichment analysis of interactions with a PPI enrichment *p*-value of < 1.0e-16 displayed a significantly higher number of interactions (edges) than expected (DMSO_IP: 89 edges; MG132_IP: 1216 edges). To elucidate interacting proteins sharing a related function or involved in the same biological pathway, the PPI networks were subdivided into 12 clusters using the clustering method k-means clustering provided by the STRING database (Figure 3C and Figure 4C; Supplementary Tables S12 and S13). Most of the densely connected proteins in the clusters were also predicted to interact with proteins of other clusters, indicating a multiple role of the BAG3 interactors in different pathways. In addition to clustered proteins, a substantial number of identified potential BAG3 interactors were not associated to any cluster of the networks, illustrating a diverse spectrum of BAG3 interactors with various functions in different pathways.

The 12 clusters found in the BAG3 PPI network under basal conditions (DMSO_IP) refer to following protein interactions, protein complexes or cellular processes (Figure 3C; Supplementary Table S12): In Cluster 1 (red nodes), proteins with no predicted interaction with another protein of the network or with only one interactor were collected. Additionally, Cluster 1 contains proteins that could not be assigned to one of the 12 generated k-means clusters, but are predicted to interact with proteins of the clusters. A small subcluster of Cluster 1 consists of the five proteins ATXN2, G3BP1, ECD4, DDX6 and STAU1, all involved in mRNA metabolism. Besides the calcyclin-binding protein CACYBP, the chaperonin HSPD1 and the tubulin family protein TUBB4B, Cluster 2 (lime green nodes) comprises the TriC (*T-complex protein ring complex*) complex proteins CCT4 and CCT5, the ribosomal protein RPL3 as well as the components EIF3E and EIF3L of the eIF-3 complex, all involved in translation and protein folding. Cluster 3 (light green nodes) was formed around the multifunctional tumor suppressor protein p53 (TP53) and contains proteins linked to the p53 pathway, such as RPA1, BAG5, TNRC6A or ANXA5. In Cluster 4 (green nodes), two components of the Sin3/HDAC complex, namely HDAC1 and SIN3A, were associated with the Sin3- or HDAC1-associated proteins SAP130 and RBBP4 as well as the transcriptional repressor proteins GATAD2A/B and corepressor CTBP2; this cluster is functionally linked to the regulation of chromatin accessibility and remodeling and thereby linked to transcriptional regulation in particular pathways. The ARP2-ARP3 complex component ACTR3 as well as proteins such as PACSIN3, GAPVD1, PLS3, CORO1C, and DCTN2 of Cluster 5 (cyan nodes) play a role in biological processes requiring actin cytoskeleton (re-)organization (e.g., endocytosis). The center protein of Cluster 6 (dark cyan) is the adaptor protein 14-3-3 beta/alpha (YWHAB) regulating a broad spectrum of signaling pathways and processes. YWHAB is connected to the importin subunit KPNA1, the GTPase RAB14 and the TSC1–TSC2 (hamartin–tuberin) complex, a negative regulator of mTORC1 (*mammalian target of rapamycin complex 1*).

In Cluster 7 (blue nodes), the ribonucleoproteins HNRNPH1 and FUS, both components of the hnRNP (*heterogeneous nuclear ribonucleoprotein*) complex, were linked to the splicing factor subunit SF3A2 and the splicing factor RBM22, all functioning in pre-mRNA processing. FUS recruited the co-chaperone DNAJC7 to this cluster. Cluster 8 (purple nodes) associates the t-RNA ligases VARS, YARS and WARS with the BTB/POZ domain-containing proteins KCTD1 and KCTD15, both potentially repressing AP-2 transcriptional activity. The core protein of Cluster 9 (pink nodes) is DKC1, a subunit of the H/ACA snoRNP (*small nucleolar ribonucleoprotein*) complex, which is connected to proteins such as SSB1, RRP1, STAU1 or the ATPase RUVBL2, all related to RNA processing. CPSF6, a component of the CFIm (*cleavage factor Im*) complex, the transcriptional repressor LRRFIP1 and the BAG family member BAG4 were dedicated to Cluster 10 (sandy brown nodes). Cluster 11 (brown nodes) represents the components of the WDR11 complex involved in endosome-to-trans-Golgi network trafficking. Cluster 12 (yellow nodes) reflects the predicted interaction of the RNA-binding factor IGF2BP2 with WFS1 and the PKA (*protein kinase A*) and PKC (*protein kinase C*) anchoring protein AKAP12, maybe based on their proposed function in insulin signaling.

Based on the relatively larger number of identified potential interactors, the PPI network of BAG3 upon proteasome inhibition is more extensive and complex; thus, the 12 constructed k-means clusters represent subnetworks of more or less densely connected proteins and of different sizes (Figure 4C; Supplementary Table S13). Therefore, only representative proteins of the respective clusters were discussed in the following, revealing the overall biological function of the corresponding cluster (for detailed function of the proteins see Supplementary Table S13). As in the BAG3 network under basal conditions, Cluster 1 (red nodes) of the BAG3 network under proteostasis stress unified a variety of proteins that either associate only with one other protein, are not connected to any protein of the network at all or are not allocated to one of the 12 constructed k-means clusters, but are supposed to interact with members of these clusters. Nevertheless, Cluster 1 comprises a larger subcluster containing potential BAG3 interactors that are implicated in cytoskeleton dynamics and organization as well as in cellular transport processes. The cytoskeletal proteins DCTN4, DCTN5 and ACTR10 (all linked to the multiprotein complex dynactin), the ATP-binding component ACTR3 of the ARP2/3 complex, the dynein intermediate chain DYNC1I2 (subunit of dynein 1 complex), the motor protein KIF4B, the coatomer subunits ARCN1/COPD and COPB2, the coatomer-binding protein SCYL1, the AP-2 (*adaptor protein*) complex subunits AP2M1 and AP2B1 as well as the CLTC protein, the heavy chain of clathrin, were found in this subcluster. Potential BAG3 interactors related to cell cycle were assembled in Cluster 2 (lime green nodes). In addition to the centrosome-associated proteins CEP55, CEP97, CEP250 and TUBGCP2, the cyclin-B proteins CCNB1 and CCNB2, the kinases PLK1, TTK and CDK11A/B, the APC/C component ANAPC7, the kinetochore protein NUF2, the kinesin KIF4A, the cytoskeletal proteins DCTN2 and TUBB4B as well as the regulatory subunit NCAPG of the condensin-2 complex are members of Cluster 2. Cluster 3 (light green nodes) contains proteins linked to the p53 pathway and proteins fulfilling a function in chromatin organization and transcriptional regulation by remodeling and providing accessibility of chromatin. Core proteins of this cluster are the cellular tumor suppressor protein p53 (TP53) and components of the Sin3/HDAC complex, namely the scaffolding protein SIN3A and the catalytic subunits HDAC1 and HDAC2. In addition to other proteins, the BAF (hSWI/SNF) complex subunits SMARCB1, SMARCD1, and ACTL6A, the histone-binding proteins RBBP4, the transcriptional repressor protein GATAD2B, and the corepressor of transcription regulators CTBP1 were numbered among this cluster. Proteins of Cluster 4 (green nodes) are involved in mRNA metabolic processes including processing, degradation and translation of mRNAs. Besides proteins regulating mRNA translation such as LARP4, LARP4B, DHX36, ATXN2 or STAU1, the multifunctional key player in mRNA metabolism PABPC1, the core stress granule protein G3BP1 and factors involved in mRNA processing or decapping/degradation, such as the components CNOT3 and CNOT10 of the CCR4-NOT complex, the RNA helicase DDX6 or the decapping proteins EDC4 and DCP1B, are part of Cluster 4. Additionally, translation initiations factors such as subunits of the eIF-3 complex (EIF3A, EIF3C, EIF3CL, EIF3D, EIF3E, EIF3L) or of the eIF-4G complex (EIF4G2, EIF4G3) as well as the ribosome subunits RPL3 and RPL28 were assigned to this cluster. Cluster 5 (cyan nodes) exhibits proteins functioning in (pre-) ribosomal RNA processing and transport, thereby also implicated in ribosome biogenesis. Among others, the RNA helicases DDX51, DDX52 and DHX33, the H/ACA snoRNP complex subunits DKC1 and NHP2, the ribosomal biogenesis factor NOC4L, the ribosome assembly factor NOB1, the exonuclease REXO4, the ribosomal RNA-processing proteins RRP1, RRP9, NOP14, BYSL, TSR1 and RBM34 as well as the ribosomal subunits RPS5 and RPS7 were dedicated to Cluster 5. Proteins of Cluster 6 (dark cyan nodes) play a role in pre-mRNA processing, especially in splicing (alternative splicing), 3′end processing, polyadenylation, and stabilization of pre-mRNA, therefore functionally related to proteins of Cluster 4. Key proteins of this cluster are the hnRNPs HNRNPH1, HNRNPH2, HNRNPL, PTBP1 and FUS that were connected to proteins such as the splicing factors U2AF2, SRSF6 and RBM22, the CFIm (*cleavage factor I*) 3′ end processing complex subunits CPSF6 and CPSF7, the RNA-binding proteins RBFOX2 and LUC7L2 as well as the intron-binding spliceosomal protein AQR. The members of Cluster 7 (blue nodes) are mainly enzymes involved in biosynthetic processes, including nucleotide biosynthesis as well as biosynthesis of amino acids and proteins. Beside the transferases GART, PPAT, APRT and ATIC, the synthetases GMPS, UMPS, TYMS, GLUL, MTR and PFAS as well as the t-RNA ligases VARS, YARS and SARS were connected to each other in Cluster 7. Potential BAG3 interactors of Cluster 8 (purple nodes) can be grouped under the broader term protein quality control. The HSP70 chaperones HSPA4 and HSPA6, the small HSP HSPB8, the nucleotide-exchange factor HSPH1 as well as the co-chaperones DNAJC7, BAG4, BAG5, and SGTA were assigned to this cluster. Cluster 9 (pink nodes) encompasses proteins linked to cellular signaling pathways, including targets, anchoring/adaptor or recruiting proteins. The tyrosine kinases SRC and YES1, the regulatory subunit PIK3R2 of the PIK3 kinase, the nuclear factor NFκB subunit p65 (RELA) or the signal transducer and transcription activator STAT1 represent members of this cluster. In Cluster 10 (sandy brown nodes), potential BAG3 interactors associated with proteasomal degradation were combined. In addition to the 26S proteasome subunits PSMB4, PSMC2, PSMC4, PSMC5, PSMD3, PSMD4 and PSMD12, the E1 enzyme UBA1, the ubiquilin UBQLN2, the proteasome-associated deubiquitinase USP14, the multiubiquitin chain receptor RAD23B and the APC/C component ANAPC5 (an ubiquitin ligase) were found in Cluster 10. Among other proteins, Cluster 11 (brown nodes) accommodates the FMRP-related proteins FXR1 and FXR2, the cytoplasmic and nuclear FMR1-interacting proteins CYFIP1, CYFIP2, and NUFIP2, as well as the RNA-binding proteins PABPC3 and PABPC4. Core protein of Cluster 12 (yellow nodes) is the subunit RPA1 of the heterotrimeric RPA (*replication protein A*) complex, recruiting the components MCM4 and MCM5 of the MCM (*minichromosome maintenance*) complex, the catalytic component POLD1 of the DNA polymerase delta complex, the core component CUL4B of the cullin-RING-based E3 ligase complex as well as the RPA-related protein RADX (CXorf57) to the cluster. All proteins can be linked to DNA replication.

In conclusion, the functional annotation and enrichment analysis as well as the PPI enrichment analysis revealed that the class A BAG3 interactomes do not fundamentally differ under basal conditions and upon proteasome inhibition. Consistently, the functional annotation and enrichment analyses of potential BAG3 interactors identified under basal (DMSO_IP) and stress conditions (MG132_IP) share a vast number of enriched GO terms in the categories Biological Process, Molecular Function and Cellular Component; herein, enriched Biological Process GO terms perfectly match with the corresponding enriched Molecular Function and Cellular Component GO terms. Furthermore, the PPI enrichment analyses and clustering of the BAG3 interactomes under basal conditions and upon proteasome inhibition are largely similar. In summary, potential BAG3 interactors identified in this study have been shown to be linked to diverse key cellular processes, including RNA metabolic processes (particularly mRNA and rRNA processing), gene expression (regulation of transcription by chromatin (re-) organization, translation), protein folding and degradation, cytoskeleton dynamics and organization, transport/trafficking, or cell cycle, and to signaling pathways, such as the p53 or SRC signaling pathways. A majority of these potential BAG3 interactors are RNA-, nucleotide- or protein-binding proteins and part of larger protein-containing complexes. In line with the already described cellular functions of BAG3, these results emphasize the multifunctionality of BAG3 and its adaptor protein function, enabling it to recruit different complexes to their site of action. Nevertheless, a more detailed examination of the interactome data set and the performed STRING analysis suggest that there are differences between the BAG3 interactomes under basal conditions and upon proteasome inhibition. Notably, while some potential BAG3 interactors were detected only under basal conditions, a much higher number of potential BAG3 binding proteins were only found upon proteasome inhibition by MG132. Under proteostasis stress, BAG3 seems to be either more or less involved in specific biological processes or even to be implicated in more diverse pathways, not least demonstrated by the larger number of significant interactors upon inhibition of proteasomal degradation. In response to cell stress, BAG3 may change its localization to specific cellular components and thus bind more or less to specific proteins. In this manner, BAG3 can actively respond to cellular proteostasis stress and is able to assist in adapting cellular homeostasis to stress.

### 3.3. Alteration in BAG3 Protein-Protein Interactions under Proteostasis Stress

**Figure 7:**
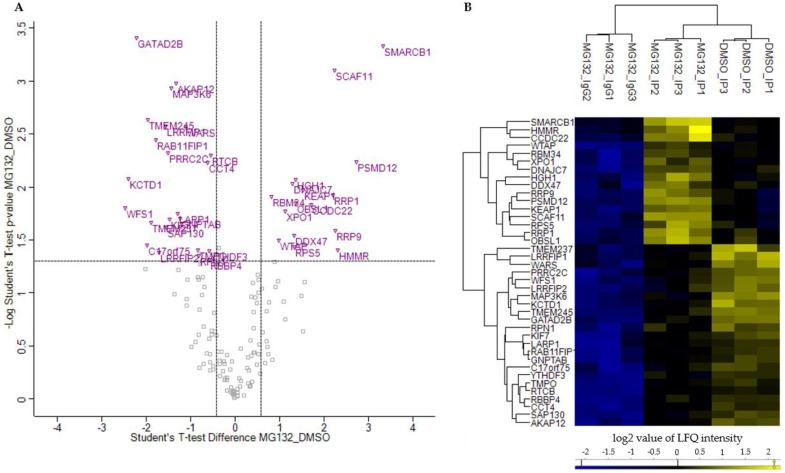
Quantitative proteomic analysis of alteration in BAG3 protein-protein interactions upon proteasome inhibition. (**A**) Scatter plot generated by plotting the log2 ratios against the negative log10 *p*-values of the Student’s t-test MG132_IP compared to DMSO_IP (MG132_DMSO; *p*-value = 0.05; S0 = 0). Proteins with a *p*-value ≤ 0.05 and a ratio ≥ 1.5 or ≤ 0.75 (black dash lines) were considered as significant, labelled with the gene symbols of the respective proteins, and are shown as purple open triangles. (**B**) Hierarchical clustering and heat map of LFQ intensities of proteins whose interaction with BAG3 was significantly changed upon proteasome inhibition. The three groups DMSO_IP, MG132_IP and MG132_IgG_IP including their three biological replicates are clustered in columns and proteins are clustered in rows. Color scale reports Z-scored log2-transformed LFQ intensity values; blue indicates a high LFQ intensity, yellow marks a low LFQ intensity. Scatter plot including Student’s t-test and hierarchical clustering/heat map were performed by Perseus software.

To determine those proteins whose interaction with BAG3 was significantly modulated upon proteasome inhibition and to quantify this modulation, three Student’s t-test (*p*-value = 0.05, S0 = 0) were performed in succession with the DMSO_IP and MG132_IP data sets. Thus, only proteins found with a *p*-value ≤ 0.05 and a ratio ≥ 1.5 in DMSO_IP as well as in MG132_IP compared to IP control MG132_IgG were considered as significant hits and further analyzed. Proteins exhibiting a *p*-value ≤ 0.05 and a ratio ≤ 0.75 or ≥ 1.5 in the two-sample analysis of MG132_IP compared to DMSO_IP were finally declared as bona fide interactors whose interaction with BAG3 was altered upon proteasome inhibition (Figure 7A, purple labelled; Supplementary Table S14). In total, we found 39 proteins whose interaction with BAG3 was significantly changed upon proteasome inhibition by MG132. In detail, 16 proteins showed an increased interaction with BAG3 (ratio ≥ 1.5) and 23 proteins a reduced interaction (ratio ≤ 0.75) upon proteasome inhibition (Table 3 and Table 4). Heat map and hierarchical clustering of the LFQ intensities of these bona fide interactors reflected their higher or lower protein abundance in DMSO_IP or in MG132_IP, respectively (Figure 7B).

**Figure 8:**
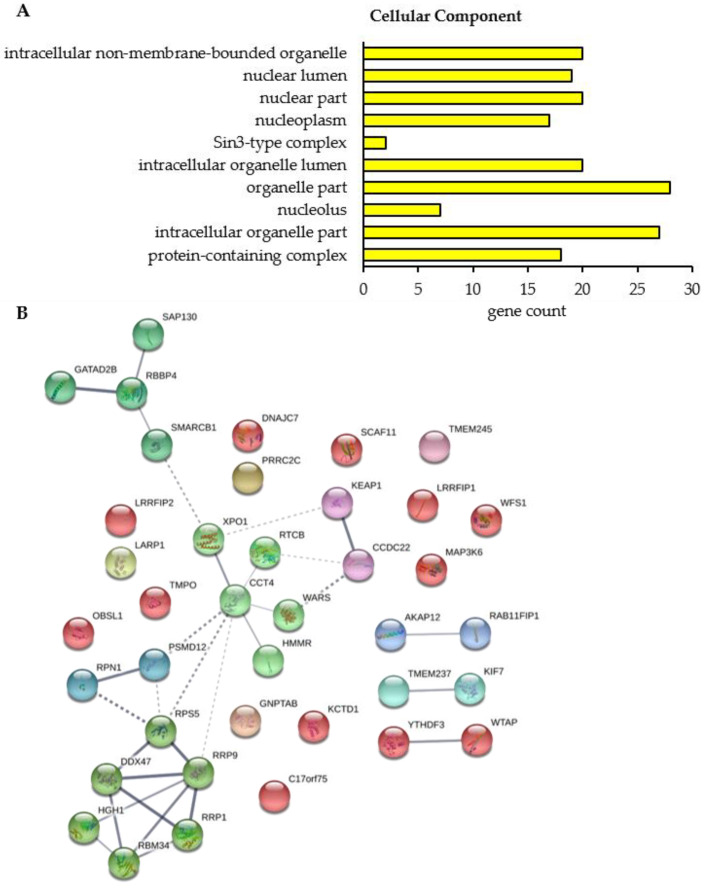
Gene ontology (GO) functional annotation/enrichment analysis and PPI enrichment analysis of proteins whose interaction with BAG3 was significantly altered upon proteasome inhibition. (**A**) Significantly enriched GO terms in the category Cellular Component are shown. For each GO term, the observed gene count is presented. (**B**) Protein-protein interaction (PPI) network of proteins whose interaction with BAG3 was significantly altered upon proteasome inhibition. Network was clustered into 12 subnetworks using the k-means clustering method; nodes/proteins of the same cluster exhibit the same color. Thickness of connecting lines/edges correlates with the strength of the association. Further network parameters: number of nodes: 39, number of edges: 31 (expected: 12), average node degree: 1.59, avg. local clustering coefficient: 0.382, PPI enrichment *p*-value: 2.39e-03, statistical background: whole genome. GO functional annotation/enrichment analysis and creating of PPI network were performed by STRING database.

To figure out the cellular function and a potential interaction between the proteins whose binding to BAG3 was modulated upon proteasome inhibition, we performed a GO functional annotation and enrichment analysis and constructed a PPI network by the STRING database. Only in the category Cellular Component, the GO functional annotation and enrichment analysis revealed 10 significantly enriched GO terms for the 39 proteins whose interaction with BAG3 was altered upon proteasome inhibition (Figure 8A; Table A7 and Supplementary Table S15). The majority of enriched Cellular Component GO terms was dedicated to the parent term nucleus, including nuclear lumen, nuclear part, nucleoplasm and nucleolus. Half of the identified proteins were functionally annotated to protein-containing complexes and thus to catalytic complexes (e.g., Sin3-type complex), in particular (Table A7).

The PPI network of the proteins whose interaction with BAG3 was changed upon proteasome inhibition is made up of 39 nodes (corresponding to proteins) and 31 edges (corresponding to interactions) (Figure 8B). The construction of this network with a PPI enrichment *p*-value of 2.39e-03 resulted in more edges/interactions than expected (12 edges). To reveal proteins possessing similar functions or participating in the same cellular processes, the PPI network was clustered into 12 subnetworks using the k-means clustering method of STRING database (Figure 8B; Supplementary Table S16). Cluster 1 (red nodes) represents a slacker cluster and encompasses a total of 12 proteins, almost all of them are not connected with another member of the network. A small subcluster was formed by the proteins WTAP and YTHDF3, both linked to N6-methyladenosine (m6A) methylation of mRNAs and thereby involved in mRNA splicing, stability and translation. WTAP as a regulatory subunit of the WMM (WTAP-METTL3-METTL14) N6-methyltransferase complex is a so called m6A writer and catalyzes the formation of m6A methylation. In contrast, the m6A reader protein YTHDF3 specifically binds to m6A-containing mRNAs and promotes their translation. In addition, the HSP70 and HSP90 co-chaperone DNAJC7, the component NJMU-R1 (C17orf75) of the WDR11 complex, the kinase MAP3K6 of the JNK signaling pathway, the transmembrane protein WFS1, participating in the regulation of cellular Ca^2+^ homeostasis, and SCAF11, regulating spliceosome assembly, were dedicated to Cluster 1. Moreover, Cluster 1 contains the lamina-associated protein TMPO, a potential player in structural nucleus organization, and the OBSL1 protein, the core component of the 3M complex regulating microtubule dynamics and genome integrity. The protein KCTD1 present in Cluster 1 has been described to repress transcriptional activity of AP-2 family members and to inhibit Wnt signal transduction pathway by β-catenin degradation. Furthermore, Cluster 1 comprises the related proteins LRRFIP1 and LRRFIP2. LRRFIP1 is implicated in diverse cellular processes in the nucleus and cytoplasm, including immune response to microorganisms and auto-immunity, remodeling of the cytoskeletal system, signal transduction pathways and transcriptional regulation of genes. The poorly studied protein LRRFIP2 has been reported to regulate TLR (*Toll-like receptor*) signaling and to potentially modulate the canonical Wnt signaling. Within Cluster 1, BAG3 binding to the proteins DNAJC7, OBSL1, SCAF11, and WTAP was detected to be enhanced upon proteasome inhibition, however the proteins MAP3K6, WFS1, NJMU-R1 (C17orf75), TMPO, KCTD1, LRRFIP1, and LRRFIP2 were found to interact less with BAG3 under proteostasis stress conditions (Table 3 and Table 4).

In Cluster 2 (lime green nodes), the DEAD box RNA helicase DDX47 is associated with the proteins RRP1, RRP9, HGH1 and RBM34 as well as with the ribosomal protein RPS5; all proteins of this cluster are linked to rRNA processing and thereby to ribosome biogenesis. The interaction of BAG3 with all these proteins was intensified upon MG132 treatment (Table 3). The core protein of Cluster 3 (light green nodes) is the molecular chaperone CCT4, a component of the chaperonin multiprotein TriC complex, which is connected to the nuclear export protein XPO1 and the t-RNA ligase WARS on one side and to the hyaluronic acid receptor HMMR and the catalytic subunit RTCB of the tRNA-splicing ligase complex on the other side. While the interaction of XPO1 and HMMR with BAG3 was increased upon proteasome inhibition, a diminished binding of BAG3 to CCT4, RTCB and WARS was determined under these conditions (Table 3 and Table 4). Cluster 4 (green nodes) was formed around the core histone-binding subunit RBBP4 that interacts with the histone deacetylase complex-associated protein SAP130, the transcriptional repressor GATAD2B and the core component of the BAF (hSWI/SNF) complex SMARCB1; all proteins are associated with chromatin remodeling and transcriptional regulation. While BAG3 associated less with SMARCB1 under proteostasis stress conditions, RBBP4, GATAD2B and SAP130 showed an enhanced binding to BAG3 upon proteasome inhibition (Table 3 and Table 4). The Clusters 5–8 each consist of two proteins. Both proteins of Cluster 5 (cyan nodes) are functionally related to ciliogenesis. The transmembrane protein TMEM237 is localized at the transition zone of cilia. The kinesin-4 family protein KIF7 represents a cilia-associated motor protein that translocates in response to activation of the Shh (*sonic hedgehog*) pathway from basal body to tip in cilia and is thereby required for ciliary formation and microtubule stability. Upon inhibition of proteasomal degradation, BAG3 bound to these proteins in a reduced manner (Table 4). Cluster 6 (dark cyan nodes) is composed of the non-ATPase regulatory subunit PSMD12 of the 26S proteasome complex and RPN1, a subunit of the membrane OST complex. The mentioned proteins are involved in protein degradation and protein modification (N-linked glycosylation), respectively. The binding of PSMD12 to BAG3 was enhanced upon MG132 treatment, however RPN1 interacted less with BAG3 upon proteasome inhibition (Table 3 and Table 4). The anchoring protein AKAP12 and the RAB11 effector protein RAB11FIP1 were unified in Cluster 7 (blue nodes). AKAP12 mediates the subcellular compartmentation of PKA (*protein kinase A*) and PKC (*protein kinase C*). RAB11FIP1 has been described to act in endosomal recycling and trafficking processes. The functional connection of both proteins might be based on their link to PKA/PKC and RAB11, involved in cellular trafficking processes such as the endosomal recycling process. A diminished interaction of both proteins with BAG3 was detected upon proteasome inhibition (Table 4). In contrast, the binding of BAG3 to the proteins KEAP1 and CCDC22 of Cluster 8 (purple nodes) was shown to be enhanced under proteostasis stress (Table 3). Both proteins function in major cellular signaling pathways. The CCDC22 protein is implicated in the regulation of NF-κB signaling by mediating the degradation of inhibitory IκB proteins and thereby activating the NF-κB signaling pathway. Additionally, it is a component of the CCC (COMMD/CCDC22/CCDC93) complex that regulates the endosomal recycling of surface proteins. KEAP1 represents a cysteine-based key sensor of oxidative and electrophilic stress. As a substrate-specific E3 ligase adaptor protein, KEAP1 ubiquitinates the transcription factor NRF2 and targets it to proteasomal degradation, thereby negatively regulating the expression of cytoprotective genes (NRF2 pathway). In response to oxidative stress, KEAP1 is inactivated and NRF2 can mediate gene expression. Moreover, KEAP1 has been reported to be sequestered by p62 (SQSTM1) under stress conditions, also leading to NRF2 activation. Clusters 9 to 12 exhibit only one protein each. Cluster 9 (pink node) contains the mostly uncharacterized transmembrane protein TMEM245 whose interaction with BAG3 was reduced under proteostasis stress (Table 4). GNPTAB of Cluster 10 was observed to bind less to BAG3 upon proteasome inhibition (Table 4). The gene *GNPTAB* encodes two subunits of the enzyme GlcNAc-1-phosphotransferase that catalyzes the transfer of GlcNAc-1-phosphate to mannose residues in the oligosaccharides of newly synthesized lysosomal hydrolases in M6P synthesis and is thereby involved in the targeting of hydrolases to lysosomes. Cluster 11 (brown nodes) consists of the protein C-terminus-binding and RNA-binding protein PRRC2C involved in formation of stress granules. Its interaction with BAG3 was reduced upon proteasome failure (Table 4). The conserved RNA-binding protein LARP1 in Cluster 12 (yellow nodes) was determined to interact to a lesser extent with BAG3 upon proteasome inhibition (Table 4). LARP1 associates in an mTOR-dependent manner with the 5′cap of mRNAs (5′ terminal oligopyrimidine (5′TOP) motif), thereby regulating the stability and/or translation of mRNAs that are essential for ribosome biogenesis, cell growth and proliferation.

Taken together, the performed quantitative BAG3 interaction profilings under basal conditions and upon proteasome inhibition by MG132 revealed that BAG3 indeed alters its interaction with specific proteins involved in diverse signaling pathways and biological processes. These processes include mRNA and rRNA metabolism, chromatin remodeling and transcriptional regulation, protein folding and quality control, cytoskeletal dynamics as well as trafficking. BAG3 showed either a significantly diminished or increased interaction with proteins implicated in these pathways. Thus, the reduced or enhanced interaction of BAG3 with these proteins could result in an inhibiting or activating effect on the protein function and therefore on the respective signaling pathway or cellular process.

### 3.4. The Tyrosine-Protein Kinase YES1 Interacts with BAG3

**Figure 9:**
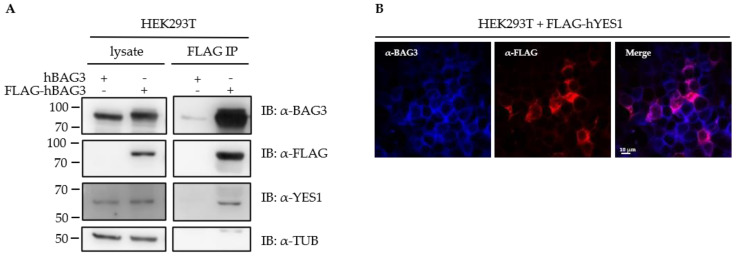
The SRC tyrosine kinase YES1 was identified as a novel BAG3 interactor. (**A**) HEK293T cells were transfected with a human BAG3 (hBAG3) or a FLAG-tagged human BAG3 (FLAG-hBAG3) overexpressing plasmid and total proteins were extracted; FLAG immunoprecipitation was performed and the eluates were separated by SDS-PAGE. Expression of indicated proteins was analyzed by immunoblotting. (**B**) HEK293T cells were transfected with a FLAG-tagged human YES1 overexpressing plasmid. Fixed cells were immunohistochemically stained with anti-BAG3 (blue) and anti-FLAG (red) and analyzed by confocal microscopy. Scale bar: 10 μm.

Recent studies reported functional interactions of the BAG3-HSP70 complex with key components of the Hippo signaling pathway such as LATS1 (*large tumor suppressor kinase 1*) and demonstrated a cross-talk between this pathway and the BAG3-mediated aggresomal targeting [30,33,72,106]. Upon proteasome inhibition, the BAG3-HSP70 complex has been shown to monitor the effectiveness of protein degradation and to activate the Hippo pathway as an adaptive response to cellular proteostasis stress [33]. We identified the tyrosine-protein kinase YES1 (*Yamaguchi Sarcoma viral oncogene homologue 1*) among the significant BAG3 interactors (Figure 4A; Supplementary Table S3). YES1 is a non-receptor protein tyrosine kinase and belongs to the SRC A family. YES1 functionally interacts with YAP1 (*Yes-associated protein 1*), that represents the downstream target of LATS1/2 in the Hippo pathway and functions as a transcriptional regulator [107]. To confirm the interaction of BAG3 with YES1, we conducted a co-immunoprecipitation assay. To that end, a FLAG-tagged version of human BAG3 (FLAG-hBAG3) was overexpressed in HEK293T cells and an anti-FLAG immunoprecipitation was performed, followed by an analysis of co-immunoprecipitated proteins via immunoblotting (Figure 9A). As control, an untagged version of BAG3 (hBAG3) was used. Endogenous YES1 co-immunoprecipitated only with FLAG-hBAG3 (not with hBAG3) (Figure 9A). To determine the subcellular distribution of BAG3 and YES1 by immunofluorescence staining, a FLAG-tagged version of human YES1 was overexpressed in HEK293T cells and fixed cells were stained with anti-BAG3 and anti-FLAG antibodies (Figure 9B). Fluorescence staining displayed a diffuse cytoplasmic localization of endogenous BAG3 and FLAG-hYES1 in HEK293T cells. In sum, the interaction of BAG3 with YES1 could be verified by co-immunoprecipitation and a diffuse cytoplasmic distribution of both proteins could be shown by immunofluorescence staining.

## 4. Discussion

The BAG3 protein represents a cellular “multiplayer”, ensuring cellular homeostasis by its cytoprotective action [16,17,18,85]. Besides its functions under basal conditions, BAG3 and BAG3-mediated selective macroautophagy have been reported to play a pivotal role in response to cellular stress and to be part of a cellular safeguarding system. Blocking of the proteasomal degradation pathway by MG132 induces cellular stress, including proteostasis stress, and thereby affects cellular signaling pathways and key processes, such as autophagy, apoptosis or cell cycle. Upon proteasome inhibition by MG132, BAG3 has been shown to be upregulated and BAG3-mediated aggresomal targeting of degradation-prone clients and their turnover by selective macroautophagy are enhanced [24,25,44,45,108].

In this study, we elucidate the multiple activities of BAG3 under basal conditions and under proteostasis stress induced by MG132. By performing qAP-MS in HEK293T cells, we established BAG3 interactomes under basal and proteostasis stress conditions (Figure 2, Figure 3 and Figure 4; Table 1 and Table 2; Supplementary Tables S1–S5) and defined proteins whose interaction with BAG3 was significantly altered upon proteasome failure (Figure 7 and Figure 8; Table 3 and Table 4; Supplementary Table S14). Using GO functional annotation/enrichment and PPI enrichment analyses, we characterized the identified BAG3 interacting proteins in respect to their involvement in biological processes, their cellular localization and their molecular functions (Figure 5, Figure 6, and Figure 8, Table A1, Table A2, Table A3, Table A4, Table A5, Table A6 and Table A7, Supplementary Tables S6–S11 and S15). The established BAG3 interactomes comprise already known and annotated BAG3 interactors (cf. annotation in BioGRID, entry BAG3) that were found in other interactome studies performed in cancer cell lines, in immortalized cells or in post-mitotic cells, such as myocytes or neurons [33,72,73,74,75,76,77]. However, we also identified several potentially novel BAG3 interactors and thus expand the existing BAG3 interactome, in particular upon proteasome inhibition. The functional annotation/enrichment and PPI enrichment analyses of BAG3 binding proteins identified in this study revealed, as expected, a vast variety of pathways and cellular processes, diverse molecular functions and different cellular components (Figure 5, Figure 6, and Figure 8, Table A1, Table A2, Table A3, Table A4, Table A5, Table A6 and Table A7, Supplementary Tables S6–S11 and S15). The higher number of potential BAG3 interactors in response to proteasome inhibition as well as the association with other cellular components (including granules or inclusion bodies) and with more diverse pathways and processes emphasize the crucial role of the multifunctional BAG3 protein under cellular stress (Figure 4 and Figure 6; Supplementary Table S3, S5, S7, S9, and S11). The BAG3 interactome under basal conditions is partly distinguished from the one upon proteasome inhibition, indicating an alteration of BAG3 binding and thereby of BAG3 function upon proteasome failure. In addition, our BAG3 interaction profiling demonstrated that binding of BAG3 to specific proteins was altered under stress in comparison to basal conditions (Figure 7 and Figure 8; Table 3 and Table 4; Supplementary Table S14). The reduced or enhanced interaction of BAG3 with respective proteins and its consequent impact on the corresponding signaling pathways or biological processes indicate that BAG3 is implicated in monitoring cellular homeostasis and in adapting the cell to stress. However, the precise effect of a diminished or increased BAG3 binding to the identified proteins and their consequences for the respective cellular signaling pathway or processes have still to be experimentally investigated and determined.

BAG3 has been described to function as a scaffolding or adaptor protein and often forms multiprotein complexes to exert its multiple activities [16,66]. Notably, components of different cellular multimeric complexes (e.g., hnRNP complexes, H/ACA RNP complexes, Sin3/HDAC complex, NuRD complex, TriC complex, dynactin complex, proteasome complex or eIF-3 complex) or proteins involved in the biogenesis or assembly of complexes were identified as BAG3 binding proteins in this study and were shown to be significantly enriched (Table A1, Table A2, Table A3, Table A4, Table A5, Table A6 and Table A7, Supplementary Tables S6–S11 and S15). As a co-chaperone the function of BAG3 in specific processes depends on its binding to the members of the HSP70/HSPA family of chaperones, especially to HSPA8 [15]. Another prominent and important interaction and complex partner of BAG3 is the small HSP protein HSPB8 [23,27,31,34,80]. In this study, several members of the HSPA family such as HSPA1, HSPA4, HSPA6 and HSPA8 as well as HSPB8 were detected as potential binding partners upon proteasome inhibition (Figure 4C, Cluster 8; Supplementary Table S3, S5, and S13). Under stress, the ternary BAG3-HSPB8-HSP70 complex is a pivotal hub for proteotoxicity-induced signaling that controls protein aggregation and forms the core complex in BAG3-mediated aggresomal targeting [24,25,33,34]. Additional factors such as 14-3-3 proteins or the HSP40 chaperone DNAJB6 are presumed to be implicated in the BAG3-triggered retrograde microtubule-based transport of clients to the aggresome [29,32]. In this study, we identified the 14-3-3 β/α protein (only under basal conditions) and the HSP40 protein family member DNAJC7 as potentially novel BAG3 interactors and detected components of the retrograde transport machinery, such as the dynein 1 complex subunits DYNC1I2 and DYNC1LI2, as well as the aggresomal marker protein vimentin (only upon proteasome inhibition) in our BAG3 interactomes (Supplementary Table S2–S5). Interestingly, binding of DNAJC7 to BAG3 was enhanced upon proteasome failure (Figure 7A; Table 3). To promote selective macroautophagy, BAG3 forms a complex with the autophagy receptor p62 (SQSTM1) and p62 was also found as a potential BAG3 interactor in this study (Supplementary Tables S2 and S3) [24].

Generally, potential BAG3 interactors identified in this study are linked to pathways and processes that have already been allocated to BAG3 and BAG3-mediated selective macroautophagy (Figure 5 and Figure 6; Table A1, Table A2, Table A3, Table A4, Table A5 and Table A6; Supplementary Tables S6–S11). Notably, proteins that altered their binding to BAG3 under proteostasis stress are also related to these pathways and processes (Figure 8A; Table A7). They perfectly match cellular functions established for BAG3 and will contribute to a better understanding of the BAG3 pathway. However, we may have additionally uncovered a linkage of BAG3 to cellular processes and signaling pathways that have yet not been associated with BAG3. In detail, processes defined for our identified potential BAG3 interactors can be assigned to the parent terms protein quality control including translation, protein folding and degradation (proteasomal and autophagic degradation), cytoskeleton dynamics and dependent processes including cellular transport/trafficking and cell cycle, signaling pathways, regulation of transcription, including chromatin remodeling, biosynthetic processes, as well as RNA metabolism.

BAG3 and the BAG3-mediated selective macroautophagy pathway play a pivotal role in cytoskeleton dynamics, organization and maintenance and thereby also impact cellular processes dependent on cytoskeletal activities, such as transport/trafficking or cell cycle [17,36,109]. In myocytes, BAG3 mediates quality control of components of the cytoskeleton and guarantees myofibrillar integrity and muscle maintenance by BAG3-mediated selective macroautophagy, here named CASA (chaperone-assisted selective autophagy) [28,30,64,72,110,111]. Moreover, BAG3 has been shown to be involved in spreading and migration in lymphoblasts as well as in motility and cell adhesion in various cancer cells [30,62,112,113,114,115]. The BAG3-mediated aggresomal targeting process towards MTOC depends on the complexing of BAG3 and dynein with the retrograde microtubule-based transport machinery [24]. In accordance with these studies, we detected BAG3 binding proteins under basal as well as under stress conditions that are linked to cytoskeleton dynamics, especially to actin and microtubule dynamics, to actin- and tubulin-based transports as well as to the MTOC (Table A1, Table A2, Table A3, Table A4, Table A5, Table A6 and Table A7; Supplementary Tables S6–S11 and S15). Exemplarily, subunits of the dynactin complex (e.g., DCTN2/p50, DCTN4/p62, ACTR10/ARP11 or CAPZA1), motor proteins of the dynein and kinesin family (e.g., DYNC1I2 or DYNC1LI2 and KIF7 or KIF4), tubulins (e.g., TUBB4B or TUBGCP2/4), and other microtubule-associated proteins, including MAP4 and DNM2, were found in the BAG3 interactomes established in this study (Figure 3C, Cluster 5 and Figure 4C, Cluster 1; Supplementary Tables S2–S5, S12 and S13). Noteworthy, components of the TriC complex mediating actin and tubulin folding were also among the identified potential BAG3 interactors (Supplementary Tables S2 and S3) [116]. Moreover, this interactome study revealed components of complexes implicated in cellular transport and trafficking, such as the coatomer complex (e.g., ARCN1 or COPB1/2), the AP-2 complex (e.g., AP2A2, AP2B1 or AP2M1) or the WDR11 complex (WDR11, FAM91A1 and C17orf75) (Figure 3C, Cluster 11 and Figure 4C, Cluster 1; Supplementary Tables S2, S3, S12, and S13), suggesting BAG3 function in endosomal trafficking. A subset of the identified potential BAG3 interactors is functionally linked to cell cycle (Figure 4C, Cluster 2; Supplementary Tables S3 and S13). In dividing cells, the chaperone complex BAG3–HSPB8 has been demonstrated to be involved in the proper spindle orientation and chromosome segregation during mitosis and in the accurate disassembly of the actin-based contractile ring during cytokinesis by affecting actin dynamics [80,81]. In line with these studies, we found centrosomal and centrosome-associated proteins (e.g., CEP55, CEP250, CEP97, CENPE, or TUBGCP2), kinetochore proteins (e.g., NUF2, PMF1 or ZNF207), cell cycle specific kinases (e.g., CDK11 or PLK1), cyclin B proteins (CCNB1/2) and components of the APC/C complex (e.g., ANAPC5 or ANAPC7) and of the MCM complex (e.g., MCM4 or MCM5) in the BAG3 interactome upon proteasome inhibition (Figure 4C, Cluster 2 and 12; Supplementary Tables S3 and S13).

Especially in response to stress, BAG3 expression and its cellular function are modulated by diverse signaling pathways; vice versa, BAG3 has been reported to sense cellular homeostasis and to transduce these signals to downstream pathways, resulting in a dynamic interplay of BAG3 and cellular signaling. A cross-talk between BAG3 and signaling networks, such as the MAPK/JNK pathway [33], the PI3K/AKT signaling pathway [69,117,118,119], the NFκ-B pathway [57,58,120,121], the SRC signaling pathway [68,77], the KEAP1-NRF2 signaling axis [31,51], and the Hippo signaling network [30,33,72], has previously been described. In this study, we identified several signaling molecules, that are part of these signaling networks, or proteins associated to these signaling pathways. For example, we found the MAPK kinase MAP3K5, the regulatory subunit PIK3R2 of PI3K, the NFκ-B transcription factor subunit p65 (RELA), the tyrosine kinase SRC, the NRF2 repressor protein KEAP1 or AMOTL2, an inhibitor of the transcription factors YAP/TAZ in the Hippo signaling, as potential BAG3 interactors (Figure 3C, Cluster 6 and 12; Figure 4C, Cluster 9; Figure 8B, Cluster 8; Supplementary Tables S2, S3, S12, S13, and S16). Intriguingly, KEAP1 was shown to enhance its interaction with BAG3 under proteostasis stress, however the binding of MAP3K5 to BAG3 was reduced under these conditions (Figure 7A; Table 3 and Table 4). Additionally, the multifunctional tumor suppressor protein p53 and several proteins linked to the p53 pathway and apoptosis were detected as potential BAG3 binding proteins under basal as well as under stress conditions (Figure 3C, Cluster 3; Figure 4C, Cluster 3; Supplementary Tables S12 and S13). Considering the well-studied anti-apoptotic function of BAG3 and the realization of this study in immortalized clonal cells (HEK293T), this finding is not particularly unexpected. However, a direct interplay of the BAG3 pathway and the p53 pathway as well as a cross-talk between autophagy and apoptosis have already been shown [40,122,123,124]. Further studies have to reveal whether BAG3 represents a dual regulator of autophagy and apoptosis, like p53, or whether its anti-apoptotic and pro-autophagic activities are rather distinct issues dependent on cellular conditions [40]. Like other cellular processes, autophagy has been demonstrated to be regulated by calcium signaling [125,126]. BAG3 is supposed to be implicated in the regulation of cellular calcium homeostasis in myocytes [127]. Intriguingly, proteins linked to the calcium signaling network, such as WFS1, WNK1, CACNA2D1 and CACYBB, were found in the BAG3 interactomes established in this study (Figure 3C and Figure 4C; Supplementary Tables S12 and S13). Its interaction with the mTOR1 inhibitors TSC1 and TSC2, both also identified as interactors in this study, links the BAG3 protein to the mTOR signaling pathway, whereby it is able to coordinate macroautophagy and protein synthesis (Figure 3C, Cluster 6; Figure 4C, Cluster 2 and 3; Supplementary Tables S12 and S13) [64]. Notably, a subgroup of the identified potential BAG3 interactors are functionally dedicated to translation/protein synthesis and associated with ribosome biogenesis (Figure 3C, Cluster 2; Figure 4C, Cluster 4 and 5; Figure 8B, Cluster 2; Supplementary Table S12, S13, and S16).

BAG3 regulates transcription of specific genes by modulating the activity of diverse transcription factors. To that end, it either affects upstream components in the corresponding signaling pathway, interacts directly with transcriptional regulators (e.g., with HSF1) or even binds to the gene promotor [33,49,55,68,73]. Besides the identification of a variety of transcriptional regulators (mostly repressors), our BAG3 interaction profiling led to the detection of proteins related to chromatin organization and regulation of chromatin accessibility by remodeling (Figure 3C and Figure 4C; Supplementary Tables S12 and S13). Under basal and stress conditions, we could find the histone deacetylase complex SIN3/HDAC and associated proteins, such as SAP130, RBBP4 or RBBP7, as well as the histone acetylase complex NuA4 (RUVBL1/2, ACTL6A/BAF53A) as potential BAG3 interacting partners (Figure 3C, Cluster 4; Figure 4C, Cluster 3; Supplementary Tables S12 and S13). A recent study revealed that BAG3 stabilizes the glutaminase GLS, leading to the promotion of glutaminolysis and thereby inducing autophagy [71]. In combination with this finding, the identification of enzymes implicated in nucleotide, amino acid and protein synthesis in this study suggests a function of BAG3 in biosynthetic processes (Figure 3C, Cluster 8; Figure 4C, Cluster 7; Supplementary Tables S12 and S13). Transferases, such as GART, PPAT, ATIC or APRT, synthetases such as GMPS, UMPS, TYMS, GLUL, MTR or ASNS and t-RNA ligases, including VARS, YARS, and SARS, were among the detected BAG3 binding proteins.

Interestingly, a majority of the identified potential BAG3 interactors is linked to RNA metabolism (Figure 3, Figure 4, Figure 5, Figure 6, Figure 7 and Figure 8; Table A1, Table A2, Table A3, Table A4, Table A5, Table A6 and Table A7; Supplementary Tables S2, S3, S6–S16). One part of this group of proteins, including RRP1, RRP9, TSR1, the RNA helicases DDX51, DDX52 and DDX47 as well as the two subunits DKC1 and NHP2 of the H/ACA snoRNP complex, is implicated in rRNA processing and transport (Figure 3C, Cluster 9; Figure 4C, Cluster 5; Figure 8B; Cluster 2; Supplementary Tables S12, S13, and S16). However, the other part of this group of proteins is involved in mRNA metabolism, including processing, translation, stabilization, and degradation of mRNAs. In detail, detected proteins such as the hnRNPs HNRNPH1/2, HNRNPL, PTBP1 and FUS, the RNA-binding proteins RBFOX1-3, SSB and YTHDF3, the splicing factors U2AF2, SRSF6 and RBM22 or the cleavage stimulation factors CSTF1/2 and CPSF6/7 mediate processing and stabilization of mRNAs (Figure 3C, Cluster 1 and 7; Figure 4C, Cluster 4 and 6; Supplementary Tables S12 and S13); identified potential BAG3 interactors such as the decapping activators EDC4, DDX6 or PATL1 and the co-activator DCP1A promote the decapping and the decay of mRNAs (Figure 3C, Cluster 1; Figure 4C, Cluster 4; Supplementary Tables S12 and S13). Detected proteins such as components of the eIF-3 complex (EIF5B, EIFG2/3, LARP4) or proteins of the large and small ribosomal subunit (RPS3, RPS5, RPL3, RPL28) can be linked to mRNA translation (Figure 4C, Cluster 4; Supplementary Table S13). Several studies demonstrated that BAG3 is able to directly interact with mRNAs, as for instance with LC3B mRNA, SKP2 mRNA, HK2 mRNA or CXC4 mRNA, and thereby stabilizing these transcripts [70,128,129,130]. An et al. has even suggested two RNA-binding domains (67–76 aa and 473–485 aa) within the BAG3 protein and additionally showed the binding of BAG3 to the snoRNP IMP3 [130]. Indeed, ribosomal and RNA-binding proteins were also found in other established BAG3 interactomes [74]. Two recent studies elucidate the function of BAG3 in granulostasis and may also explain the large number of identified potential BAG3 interactors linked to RNA metabolism [33,34]. In cells, ribonucleoprotein (RNP) granules such as stress granules (SGs) or processing bodies (P-bodies) are formed under stress to store or degrade mRNAs (e.g., upon proteasome inhibition by MG132) [131,132,133,134]. SGs are highly dynamic; depending on cellular state, they can contain different components, including defective ribosomal products (DRiPs), poly(A) mRNAs, RNA-binding proteins, 40S ribosomal subunits, transcription elongation factors such as eIF2, eIF3, eIF4E, eIF4G, eIF4A, or eIF4B, components of the ubiquitin–proteasome system and autophagy factors [135,136]. The HSPB8-BAG3-HSP70 complex has been reported to monitor the formation of DRiPs in SGs in concert with components of the ribosome-associated quality control system and the ribosome-associated chaperone complex NAC (*nascent polypeptide-associated complex*), to sequester DRiPs to the aggresome and thus to target them together with p62/SQSTM1 to autophagic degradation [33,34]. In this manner, BAG3 helps to prevent accumulation of DRiPs and other misfolded proteins in SGs and maintains the composition, dynamics and disassembly kinetics of SGs (granulostasis), guaranteeing proper translation [34]. Moreover, Mateju et al. has supposed that aggresome formation is essential to link clearance of aberrant SGs to autophagy [137]. Intriguingly, a detailed analysis of the BAG3 interactomes established in this study revealed not only SG and P-body marker proteins (such as PABP1, G3BP10 and ATXN2 or DCP1A, EDC4, DDX6, LSM14A, and PATL1) among our potential BAG3 interactors, but also showed that almost all detected proteins associated with mRNA metabolism can be found in SGs and P-bodies (Figure 3, Figure 4, Figure 5 and Figure 6; Figure 3C, Cluster 1; Figure 4C, Cluster 4 and 6; Tables S2–S13) (cf. SG database https://msgp.pt) [136].

Among the significant BAG3 interactors, we identified the kinase YES1 as a potential BAG3 binding protein (Figure 4; Supplementary Table S3). We verified its interaction with BAG3 by co-immunoprecipitation and showed a diffuse cytoplasmic distribution of both proteins by immunofluorescence staining (Figure 9). Like SRC, YES1 is a non-receptor tyrosine-protein kinase of the SRC kinase family and has been described to be involved in different processes, such as cell proliferation, apoptosis or cytoskeleton remodeling. In complex with HSP70, BAG3 has already been shown to interact with the SH3 domain of SRC via its PxxP region, thus affecting SRC signaling pathway in cancer cells [68,77]. YES1 has been reported to interact with YAP/YAP1 [107]. By phosphorylation of YAP, YES1 regulates the formation, localization and activity of the YAP-β-Catenin-TBX5 complex, thereby promoting the survival and transformation of β-catenin-active cancer cell lines [107,138]. Furthermore, YAP acts as a transcriptional co-activator in the Hippo signaling pathway. Within the Hippo signaling cascade, the activity of YAP is regulated by the Hippo kinases LATS1/2 and STK38 as well as by proteins of the angiomotin (AMOT) family, e.g., AMOTL1/2 [139,140,141]. Previous studies uncovered an interaction of LATS1/2 and STK38 with BAG3 and demonstrated a bi-directional cross-talk between the Hippo network and the BAG3 pathway [30,72]. Under proteotoxic stress, the BAG3-HSP70-LATS1 signaling axis is involved in the regulation of protein aggregation [33]. Interestingly, YES1 has also been reported to be associated with SGs (cf. SG database https://msgp.pt) [136]. Considering these studies, the identified BAG3 interacting protein YES1 may represent a further signaling node in the regulation of BAG3 function and the BAG3-mediated selective macroautophagy pathway. The functional relevance of this BAG3–YES1 interaction remains to be determined and studied in detail. Since BAG3 has been shown to be a phosphoprotein and its phosphorylation impacts its cellular function, a possible phosphorylation of BAG3 by the tyrosine kinase YES1 has also to be considered and examined [16].

## 5. Conclusions

In conclusion, BAG3 exerts diverse functions in cellular protein and organelle quality control, thereby ensuring cellular homeostasis. To that end, it affects folding, sequestration, and autophagic degradation of proteins, coordinates transcription, translation, and degradation, and also modulates respective signaling pathways. By complexing with other proteins and targeting them to their site of action, BAG3 represents a pivotal player in the cellular adaptation process in response to stress. This study expands the spectrum of potential BAG3 interactors and elucidates the multifunctionality of BAG3 as well as its crucial role in proteostasis, thus contributing to a better understanding of BAG3 function and the associated processes and pathways. Regarding its proven implication in severe diseases such as cancer, myopathies and neurodegenerative disorders, the herein revealed new aspects of BAG3 function and the BAG3 pathway emphasize the potential of BAG3 as a therapeutic target.

## Figures and Tables

**Table 1:** Top 25 of enriched class A BAG3 interactors (*p*-value ≤ 0.01 and ratio ≥ 2) identified under basal conditions (DMSO_IP).

Majority Protein ID	Gene Name	Protein Name	*p*-Value	Ratio
Q9UL15	BAG5	BAG family molecular chaperone regulator 5	1.08 × 10^−4^	503.14
Q6WKZ4	RAB11FIP1	Rab11 family-interacting protein 1	2.77 × 10^−3^	321.23
Q9HAS0	C17orf75	Protein Njmu-R1	4.50 × 10^−3^	145.58
Q96SI1	KCTD15	BTB/POZ domain-containing protein KCTD15	5.77 × 10^−3^	112.74
Q658Y4	FAM91A1	Protein FAM91A1	7.15 × 10^−3^	95.84
Q13547	HDAC1	Histone deacetylase 1	5.76 × 10^−4^	83.85
Q9BTA9	WAC	WW domain-containing adapter protein with coiled-coil	1.03 × 10^−3^	77.47
Q9H4A3	WNK1	Serine/threonine-protein kinase WNK1	2.58 × 10^−3^	72.78
Q9H0E3	SAP130	Histone deacetylase complex subunit SAP130	1.13 × 10^−4^	69.35
Q96KG9	SCYL1	N-terminal kinase-like protein	3.19 × 10^−3^	58.40
Q2M1P5	KIF7	Kinesin-like protein KIF7	6.33 × 10^−3^	54.73
P31946	YWHAB	14-3-3 protein beta/alpha	3.87 × 10^−4^	53.44
O76024	WFS1	Wolframin	9.50 × 10^−4^	52.92
P13797	PLS3	Plastin-3	6.69 × 10^−5^	51.70
Q3T906	GNPTAB	N-acetylglucosamine-1-phosphotransferase subunits alpha/beta	3.69 × 10^−3^	51.00
Q9Y383	LUC7L2	Putative RNA-binding protein Luc7-like 2	8.03 × 10^−4^	50.63
Q8WXI9	GATAD2B	Transcriptional repressor p66-beta	1.23 × 10^−4^	47.77
Q96ST3	SIN3A	Paired amphipathic helix protein Sin3a	8.73 × 10^−3^	39.43
Q719H9	KCTD1	BTB/POZ domain-containing protein KCTD1	9.06 × 10^−4^	38.95
P27694	RPA1	Replication protein A 70 kDa DNA-binding subunit	6.47 × 10^−5^	38.49
Q9Y3I0	RTCB	tRNA-splicing ligase RtcB homolog	1.71 × 10^−5^	37.36
Q02790	FKBP4	Peptidyl-prolyl cis-trans isomerase FKBP4	4.28 × 10^−3^	32.74
Q9H330	TMEM245	Transmembrane protein 245	1.17 × 10^−6^	32.20
P48643	CCT5	T-complex protein 1 subunit epsilon	9.87 × 10^−3^	31.30
P26196	DDX6	Probable ATP-dependent RNA helicase DDX6	9.11 × 10^−4^	30.30

**Table 2:** Top 25 of enriched class A BAG3 interactors (*p*-value ≤ 0.01 and ratio ≥ 2) identified upon proteasome inhibition by MG132 (MG132_IP).

Majority Protein ID	Gene Name	Protein Name	*p*-Value	Ratio
Q9UL15	BAG5	BAG family molecular chaperone regulator 5	1.82 × 10^−4^	335.15
Q96I24	FUBP3	Far upstream element-binding protein 3	8.54 × 10^−3^	109.91
Q01664	TFAP4	Transcription factor AP-4	5.49 × 10^−6^	109.80
Q6WKZ4	RAB11FIP1	Rab11 family-interacting protein 1	6.23 × 10^−3^	94.13
Q13547	HDAC1	Histone deacetylase 1	2.47 × 10^−4^	87.50
Q9NZ32	ACTR10	Actin-related protein 10	2.10 × 10^−3^	67.55
Q3MHD2	LSM12	Protein LSM12 homolog	7.88 × 10^−4^	57.16
Q9H4A3	WNK1	Serine/threonine-protein kinase WNK1	1.28 × 10^−3^	51.93
Q9Y383	LUC7L2	Putative RNA-binding protein Luc7-like 2	9.36 × 10^−4^	49.12
O43242	PSMD3	26S proteasome non-ATPase regulatory subunit 3	6.78 × 10^−4^	43.81
Q13283	G3BP1	Ras GTPase-activating protein-binding protein 1	2.15 × 10^−4^	43.58
P26196	DDX6	Probable ATP-dependent RNA helicase DDX6	2.07 × 10^−4^	42.44
P62195	PSMC5	26S protease regulatory subunit 8	1.38 × 10^−3^	42.30
Q99613	EIF3C	Eukaryotic translation initiation factor 3 subunit C	1.51 × 10^−3^	40.14
Q9BZD4	NUF2	Kinetochore protein Nuf2	1.05 × 10^−5^	39.23
Q9Y262	EIF3L	Eukaryotic translation initiation factor 3 subunit L	3.61 × 10^−3^	38.43
P13797	PLS3	Plastin-3	2.51 × 10^−4^	37.65
Q99615	DNAJC7	DnaJ homolog subfamily C member 7	1.55 × 10^−4^	36.01
P15104	GLUL	Glutamine synthetase	5.95 × 10^−3^	32.62
Q7KZF4	SND1	Staphylococcal nuclease domain-containing protein 1	7.04 × 10^−5^	32.33
P27694	RPA1	Replication protein A 70 kDa DNA-binding subunit	2.28 × 10^−4^	32.19
P62081	RPS7	40S ribosomal protein S7	2.89 × 10^−3^	30.79
P12814	ACTN1	Alpha-actinin-1	5.19 × 10^−5^	30.13
Q96KG9	SCYL1	N-terminal kinase-like protein	4.28 × 10^−4^	29.49
P08754	GNAI3	Guanine nucleotide-binding protein G(k) subunit alpha	1.03 × 10^−4^	28.21

**Table 3:** Proteins whose interaction with BAG3 was enhanced upon proteasome inhibition.

Majority Protein ID	Gene Name	Protein Name	*p*-Value	Ratio
Q12824	SMARCB1	SWI/SNF-related matrix-associated actin-dependent regulator of chromatin subfamily B member 1	4.70 × 10^−4^	9.98
O00232	PSMD12	26S proteasome non-ATPase regulatory subunit 12	5.83 × 10^−3^	6.60
O75330	HMMR	Hyaluronan mediated motility receptor	3.98 × 10^−2^	4.93
O43818	RRP9	U3 small nucleolar RNA-interacting protein 2	2.60 × 10^−2^	4.75
Q99590	SCAF11	Protein SCAF11	7.97 × 10^−4^	4.69
P56182	RRP1	Ribosomal RNA processing protein 1 homolog A	1.19 × 10^−2^	4.61
O60826	CCDC22	Coiled-coil domain-containing protein 22	1.47 × 10^−2^	3.26
Q14145	KEAP1	Kelch-like ECH-associated protein 1	1.08 × 10^−2^	2.88
O75147	OBSL1	Obscurin-like protein 1	1.44 × 10^−2^	2.58
Q9BTY7	HGH1	Protein HGH1 homolog	8.53 × 10^−3^	2.57
P46782	RPS5	40S ribosomal protein S5	3.69 × 10^−3^	2.50
Q9H0S4	DDX47	Probable ATP-dependent RNA helicase DDX47	2.91 × 10^−2^	2.49
Q99615	DNAJC7	DnaJ homolog subfamily C member 7	9.41 × 10^−3^	2.45
O14980	XPO1	Exportin-1	1.70 × 10^−2^	2.18
Q15007	WTAP	Pre-mRNA-splicing regulator WTAP	3.21 × 10^−2^	1.98
P42696	RBM34	RNA-binding protein 34	1.25 × 10^−2^	1.77

**Table 4:** Proteins whose interaction with BAG3 was reduced upon proteasome inhibition.

Majority Protein ID	Gene Name	Protein Name	*p*-Value	Ratio
O76024	WFS1	Wolframin	1.60 × 10^−2^	0.18
Q719H9	KCTD1	BTB/POZ domain-containing protein KCTD1	8.39 × 10^−3^	0.19
Q8WXI9	GATAD2B	Transcriptional repressor p66-beta	3.96 × 10^−4^	0.22
Q9HAS0	C17orf75	Protein Njmu-R1	3.56 × 10^−2^	0.25
Q9H330	TMEM245	Transmembrane protein 245	2.32 × 10^−3^	0.26
Q96Q45	TMEM237	Transmembrane protein 237	2.17 × 10^−2^	0.27
Q6WKZ4	RAB11FIP1	Rab11 family-interacting protein 1	3.61 × 10^−3^	0.29
Q9Y608	LRRFIP2	Leucine-rich repeat flightless-interacting protein 2	4.21 × 10^−2^	0.31
Q32MZ4	LRRFIP1	Leucine-rich repeat flightless-interacting protein 1	2.67 × 10^−3^	0.34
Q9H0E3	SAP130	Histone deacetylase complex subunit SAP130	2.41 × 10^−2^	0.34
Q9Y520	PRRC2C	Protein PRRC2C	4.83 × 10^−3^	0.35
Q2M1P5	KIF7	Kinesin-like protein KIF7	2.04 × 10^−2^	0.36
O95382	MAP3K6	Mitogen-activated protein kinase kinase kinase 6	1.17 × 10^−3^	0.37
Q02952	AKAP12	A-kinase anchor protein 12	1.05 × 10^−3^	0.40
Q6PKG0	LARP1	La-related protein 1	1.80 × 10^−2^	0.41
Q3T906	GNPTAB	N-acetylglucosamine-1-phosphotransferase subunits alpha/beta	2.02 × 10^−2^	0.43
P23381	WARS	Tryptophan--tRNA ligase, cytoplasmic	2.72 × 10^−3^	0.46
P42167	TMPO	Lamina-associated polypeptide 2, isoforms beta/gamma	4.00 × 10^−2^	0.56
P04843	RPN1	Dolichyl-diphosphooligosaccharide-protein glycosyltransferase subunit 1	4.53 × 10^−2^	0.57
P50991	CCT4	T-complex protein 1 subunit delta	5.95 × 10^−3^	0.65
Q09028	RBBP4	Histone-binding protein RBBP4	4.88 × 10^−2^	0.67
Q7Z739	YTHDF3	YTH domain-containing family protein 3	4.06 × 10^−2^	0.67
Q9Y3I0	RTCB	tRNA-splicing ligase RtcB homolog	5.03 × 10^−3^	0.68

## Supplementary Materials

The following are available online at https://www.mdpi.com/2073-4409/9/11/2416/s1, Figure S1: Histogram of processed data after imputation (Perseus), Table S1: Data of all identified proteins after processing by Perseus software, Figure S2: Validation of performed affinity purification of endogenous BAG3 within the qAP-MS approach, Table S2: DMSO_IP: Identified BAG3 interactors (*p*-value ≤ 0.05 and ratio ≥ 1.5), Table S3: MG132_IP: Identified BAG3 interactors (*p*-value ≤ 0.05 and ratio ≥ 1.5), Table S4: DMSO_IP: Identified class A BAG3 interactors (*p*-value ≤ 0.01 and ratio ≥ 2), Table S5: MG132_IP: Identified class A BAG3 interactors (*p*-value ≤ 0.01 and ratio ≥ 2), Table S6: DMSO_IP: Biological Process GO terms, Table S7: MG132_IP: Biological Process GO terms, Table S8: DMSO_IP: Molecular Function GO terms, Table S9: MG132_IP: Molecular Function GO terms, Table S10: DMSO_IP: Cellular Component GO terms, Table S11: MG132_IP: Cellular Component GO terms, Table S12: DMSO_IP: k-means clusters of PPI network, Table S13: MG132_IP: k-means clusters of PPI network, Table S14: Identified proteins that changed interaction with BAG3 upon proteasome inhibition (*p*-value ≤ 0.05 and ratio ≥ 1.5 or ≤ 0.75), Table S15: Enriched GO terms in the category Cellular Component for identified proteins that changed interaction with BAG3 upon proteasome inhibition, Table S16: Identified proteins that changed interaction with BAG3 upon proteasome inhibition: k-means clusters of PPI network.

## Appendix A

**Table A1:** The top 25 enriched GO terms related to Biological Process for proteins that significantly interact with BAG3 under basal conditions (DMSO_IP).

#Term ID	Term Description	Count	FDR
GO:0006457	protein folding	10	9.20 × 10^−5^
GO:0009892	negative regulation of metabolic process	33	9.20 × 10^−5^
GO:0010605	negative regulation of macromolecule metabolic process	32	9.20 × 10^−5^
GO:0016071	mRNA metabolic process	15	9.20 × 10^−5^
GO:0022607	cellular component assembly	29	9.20 × 10^−5^
GO:0022613	ribonucleoprotein complex biogenesis	13	9.20 × 10^−5^
GO:0022618	ribonucleoprotein complex assembly	10	9.20 × 10^−5^
GO:0034622	cellular protein-containing complex assembly	17	9.20 × 10^−5^
GO:0044085	cellular component biogenesis	31	9.20 × 10^−5^
GO:0060255	regulation of macromolecule metabolic process	51	9.20 × 10^−5^
GO:0065003	protein-containing complex assembly	23	9.20 × 10^−5^
GO:0090304	nucleic acid metabolic process	38	2.40 × 10^−4^
GO:0033036	macromolecule localization	27	3.20 × 10^−4^
GO:0051171	regulation of nitrogen compound metabolic process	48	3.50 × 10^−4^
GO:1900034	regulation of cellular response to heat	5	3.80 × 10^−4^
GO:0031324	negative regulation of cellular metabolic process	28	4.00 × 10^−4^
GO:0080090	regulation of primary metabolic process	48	5.90 × 10^−4^
GO:0010608	posttranscriptional regulation of gene expression	11	6.20 × 10^−4^
GO:0031323	regulation of cellular metabolic process	48	8.80 × 10^−4^
GO:0051172	negative regulation of nitrogen compound metabolic process	26	8.80 × 10^−4^
GO:0016070	RNA metabolic process	33	1.00 × 10^−3^
GO:0032268	regulation of cellular protein metabolic process	27	1.00 × 10^−3^
GO:0032269	negative regulation of cellular protein metabolic process	16	1.10 × 10^−3^
GO:0061077	chaperone-mediated protein folding	5	1.10 × 10^−3^
GO:0010629	negative regulation of gene expression	21	1.30 × 10^−3^

**Table A2:** The top 25 enriched GO terms related to Biological Process for proteins that significantly interact with BAG3 upon proteasome inhibition (MG132_IP).

#Term ID	Term Description	Count	FDR
GO:0044085	cellular component biogenesis	104	1.92 × 10^−15^
GO:0034641	cellular nitrogen compound metabolic process	154	1.08 × 10^−13^
GO:0022613	ribonucleoprotein complex biogenesis	37	3.31 × 10^−13^
GO:0006396	RNA processing	51	1.07 × 10^−12^
GO:0090304	nucleic acid metabolic process	127	1.10 × 10^−12^
GO:0006139	nucleobase-containing compound metabolic process	139	1.25 × 10^−12^
GO:0016071	mRNA metabolic process	45	1.88 × 10^−12^
GO:0010468	regulation of gene expression	137	4.23 × 10^−12^
GO:0043170	macromolecule metabolic process	190	5.41 × 10^−12^
GO:0046483	heterocycle metabolic process	140	5.80 × 10^−12^
GO:0060255	regulation of macromolecule metabolic process	165	8.33 × 10^−12^
GO:0006725	cellular aromatic compound metabolic process	140	9.33 × 10^−12^
GO:0010467	gene expression	119	1.33 × 10^−11^
GO:0006807	nitrogen compound metabolic process	203	1.80 × 10^−11^
GO:0010608	posttranscriptional regulation of gene expression	35	1.80 × 10^−11^
GO:0019222	regulation of metabolic process	171	2.97 × 10^−11^
GO:0044403	symbiont process	42	2.97 × 10^−11^
GO:0044265	cellular macromolecule catabolic process	48	3.22 × 10^−11^
GO:1901360	organic cyclic compound metabolic process	142	3.46 × 10^−11^
GO:0016032	viral process	39	3.79 × 10^−11^
GO:0016070	RNA metabolic process	111	3.79 × 10^−11^
GO:0022607	cellular component assembly	87	3.79 × 10^−11^
GO:0044238	primary metabolic process	209	3.79 × 10^−11^
GO:0051171	regulation of nitrogen compound metabolic process	158	3.79 × 10^−11^
GO:0071704	organic substance metabolic process	214	4.12 × 10^−11^

**Table A3:** The top 25 enriched GO terms related to Molecular Function for proteins that significantly interact with BAG3 under basal conditions (DSMO_IP).

#Term ID	Term Description	Count	FDR
GO:0003723	RNA binding	23	1.95 × 10^−9^
GO:1901363	heterocyclic compound binding	49	7.94 × 10^−6^
GO:0097159	organic cyclic compound binding	49	8.63 × 10^−6^
GO:0005515	protein binding	55	9.97 × 10^−6^
GO:0005488	binding	77	1.79 × 10^−5^
GO:0003729	mRNA binding	9	2.69 × 10^−5^
GO:0003676	nucleic acid binding	34	1.20 × 10^−4^
GO:0003730	mRNA 3′-UTR binding	5	5.60 × 10^−4^
GO:0008092	cytoskeletal protein binding	15	5.60 × 10^−4^
GO:0019899	enzyme binding	25	5.60 × 10^−4^
GO:0030554	adenyl nucleotide binding	20	5.60 × 10^−4^
GO:0051082	unfolded protein binding	6	5.60 × 10^−4^
GO:0003725	double-stranded RNA binding	5	6.60 × 10^−4^
GO:0017076	purine nucleotide binding	22	7.90 × 10^−4^
GO:0060590	ATPase regulator activity	4	9.60 × 10^−4^
GO:0000166	nucleotide binding	23	1.40 × 10^−3^
GO:0051087	chaperone binding	5	2.40 × 10^−3^
GO:0044877	protein-containing complex binding	14	2.70 × 10^−3^
GO:0032559	adenyl ribonucleotide binding	18	3.10 × 10^−3^
GO:0047485	protein N-terminus binding	5	3.10 × 10^−3^
GO:0032555	purine ribonucleotide binding	20	4.10 × 10^−3^
GO:0048027	mRNA 5′-UTR binding	3	4.10 × 10^−3^
GO:0044183	protein folding chaperone	3	4.20 × 10^−3^
GO:0005524	ATP binding	17	4.80 × 10^−3^
GO:0035639	purine ribonucleoside triphosphate binding	19	6.30 × 10^−3^

**Table A4:** The top 25 enriched GO terms related to Molecular Function for proteins that significantly interact with BAG3 upon proteasome inhibition (MG132_IP).

#Term ID	Term Description	Count	FDR
GO:0003723	RNA binding	65	9.02 × 10^−22^
GO:1901363	heterocyclic compound binding	159	4.01 × 10^−15^
GO:0097159	organic cyclic compound binding	159	1.09 × 10^−14^
GO:0005488	binding	261	9.22 × 10^−14^
GO:0003676	nucleic acid binding	112	1.48 × 10^−12^
GO:0003729	mRNA binding	23	1.52 × 10^−10^
GO:0005515	protein binding	162	3.97 × 10^−8^
GO:0004386	helicase activity	17	1.09 × 10^−7^
GO:0090079	translation regulator activity, nucleic acid binding	14	2.79 × 10^−7^
GO:0003725	double-stranded RNA binding	12	4.77 × 10^−7^
GO:0045182	translation regulator activity	15	4.77 × 10^−7^
GO:0017076	purine nucleotide binding	63	1.85 × 10^−6^
GO:0030554	adenyl nucleotide binding	55	1.86 × 10^−6^
GO:0000166	nucleotide binding	68	1.87 × 10^−6^
GO:0008135	translation factor activity, RNA binding	12	1.87 × 10^−6^
GO:0016462	pyrophosphatase activity	37	1.87 × 10^−6^
GO:0017111	nucleoside-triphosphatase activity	36	1.87 × 10^−6^
GO:0032555	purine ribonucleotide binding	61	4.46 × 10^−6^
GO:0032559	adenyl ribonucleotide binding	53	5.12 × 10^−6^
GO:0042623	ATPase activity, coupled	21	5.12 × 10^−6^
GO:0005048	signal sequence binding	9	6.50 × 10^−6^
GO:0035639	purine ribonucleoside triphosphate binding	59	6.50 × 10^−6^
GO:0005524	ATP binding	51	8.43 × 10^−6^
GO:0003743	translation initiation factor activity	9	9.46 × 10^−6^
GO:0019899	enzyme binding	67	1.08 × 10^−5^

**Table A5:** The top 25 enriched GO terms related to Cellular Component for proteins that significantly interact with BAG3 under basal conditions (DMSO_IP).

#Term ID	Term Description	Count	FDR
GO:0005829	cytosol	55	3.57 × 10^−10^
GO:0032991	protein-containing complex	52	3.75 × 10^−9^
GO:0043232	intracellular non-membrane-bounded organelle	42	3.13 × 10^−6^
GO:0005634	nucleus	57	4.26 × 10^−6^
GO:1990904	ribonucleoprotein complex	17	5.45 × 10^−6^
GO:0043226	organelle	79	1.26 × 10^−5^
GO:0043229	intracellular organelle	78	1.38 × 10^−5^
GO:0000118	histone deacetylase complex	6	2.33 × 10^−5^
GO:0005622	intracellular	84	3.82 × 10^−5^
GO:0044424	intracellular part	83	4.02 × 10^−5^
GO:0070822	Sin3-type complex	4	4.02 × 10^−5^
GO:0044451	nucleoplasm part	18	5.22 × 10^−5^
GO:0017053	transcriptional repressor complex	6	7.19 × 10^−5^
GO:0005856	cytoskeleton	25	1.20 × 10^−4^
GO:0005737	cytoplasm	72	1.30 × 10^−4^
GO:0044428	nuclear part	39	1.60 × 10^−4^
GO:0016604	nuclear body	14	1.70 × 10^−4^
GO:0031981	nuclear lumen	37	1.70 × 10^−4^
GO:0043231	intracellular membrane-bounded organelle	67	4.30 × 10^−4^
GO:1902494	catalytic complex	18	4.30 × 10^−4^
GO:0016580	Sin3 complex	3	5.50 × 10^−4^
GO:0005654	nucleoplasm	32	6.20 × 10^−4^
GO:0043227	membrane-bounded organelle	70	7.00 × 10^−4^
GO:0044444	cytoplasmic part	62	7.20 × 10^−4^
GO:0016581	NuRD complex	3	8.60 × 10^−4^

**Table A6:** The top 25 enriched GO terms related to Cellular Component for proteins that significantly interact with BAG3 upon proteasome inhibition (MG132_IP).

#Term ID	Term Description	Count	FDR
GO:0005829	cytosol	195	8.46 × 10^−38^
GO:0032991	protein-containing complex	191	1.11 × 10^−37^
GO:0043232	intracellular non-membrane-bounded organelle	157	2.80 × 10^−27^
GO:0044424	intracellular part	306	3.48 × 10^−27^
GO:0005634	nucleus	211	1.07 × 10^−26^
GO:0031981	nuclear lumen	153	3.91 × 10^−25^
GO:0044428	nuclear part	159	9.89 × 10^−25^
GO:0005654	nucleoplasm	134	5.29 × 10^−22^
GO:0043229	intracellular organelle	276	7.59 × 10^−20^
GO:0005737	cytoplasm	263	1.62 × 10^−19^
GO:0043226	organelle	278	2.30 × 10^−19^
GO:0070013	intracellular organelle lumen	163	6.16 × 10^−19^
GO:0044446	intracellular organelle part	226	2.67 × 10^−18^
GO:1990904	ribonucleoprotein complex	55	5.96 × 10^−18^
GO:0043231	intracellular membrane-bounded organelle	246	2.72 × 10^−17^
GO:0044422	organelle part	227	3.30 × 10^−17^
GO:0043227	membrane-bounded organelle	256	3.42 × 10^−16^
GO:0044444	cytoplasmic part	228	6.92 × 10^−16^
GO:0044464	cell part	310	4.36 × 10^−14^
GO:0005730	nucleolus	51	2.18 × 10^−12^
GO:0015630	microtubule cytoskeleton	52	5.39 × 10^−10^
GO:0005694	chromosome	47	7.60 × 10^−10^
GO:0044427	chromosomal part	43	9.48 × 10^−10^
GO:0035770	ribonucleoprotein granule	21	2.42 × 10^−9^
GO:0044430	cytoskeletal part	60	1.22 × 10^−8^

**Table A7:** Enriched GO terms related to Cellular Component for proteins whose interaction with BAG3 was altered upon proteasome inhibition.

#Term ID	Term Description	Count	FDR
GO:0043232	intracellular non-membrane-bounded organelle	20	4.00 × 10^−3^
GO:0031981	nuclear lumen	19	4.90 × 10^−3^
GO:0044428	nuclear part	20	4.90 × 10^−3^
GO:0005654	nucleoplasm	17	6.10 × 10^−3^
GO:0070822	Sin3-type complex	2	1.55 × 10^−2^
GO:0070013	intracellular organelle lumen	20	2.36 × 10^−2^
GO:0044422	organelle part	28	2.61 × 10^−2^
GO:0005730	nucleolus	7	4.08 × 10^−2^
GO:0044446	intracellular organelle part	27	4.08 × 10^−2^
GO:0032991	protein-containing complex	18	4.17 × 10^−2^
