# Supplementary material for: BAG3 Proteomic Signature under Proteostasis Stress
Source: Cells. 2020 Nov 4;9(11):2416. doi: 10.3390/cells9112416 (PMC7694386; doi:10.3390/cells9112416)
Supplement: Supplementary file 1 [file cells-09-02416-s001.zip › Supplements/Suppl. Figures S1 and S2.docx]

**
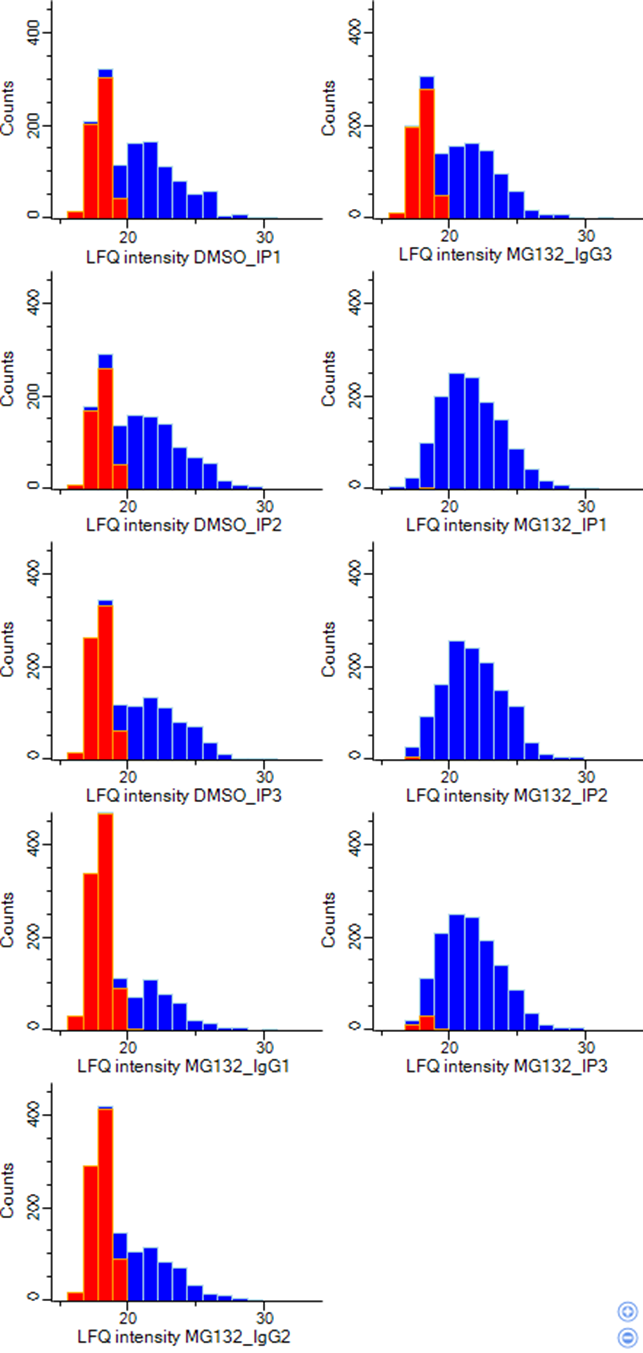
**

**Figure S1**. Histogram of processed data after imputation. DMSO_IP1-DMSO_IP3 refer to the three independent biological replicates of the BAG3 IP samples generated from DMSO-treated cells, MG132_IP1-MG132_IP3 refer to the three independent biological replicates of the BAG3 IP samples generated from MG132-treated cells and MG132_IgG1-MG132_IgG3 refer to the three independent biological replicates of the IgG IP samples generated from MG132-treated cells. In Perseus software, missing LFQ intensities of logarithmized, filtered and grouped data were replaced by low values from normal distribution (width ₌ 0.3; down shift ₌ 1.8) and a histogram of this data set was generated. Measured LFQ intensity values are colored in blue, imputed LFQ intensity values are colored in red.


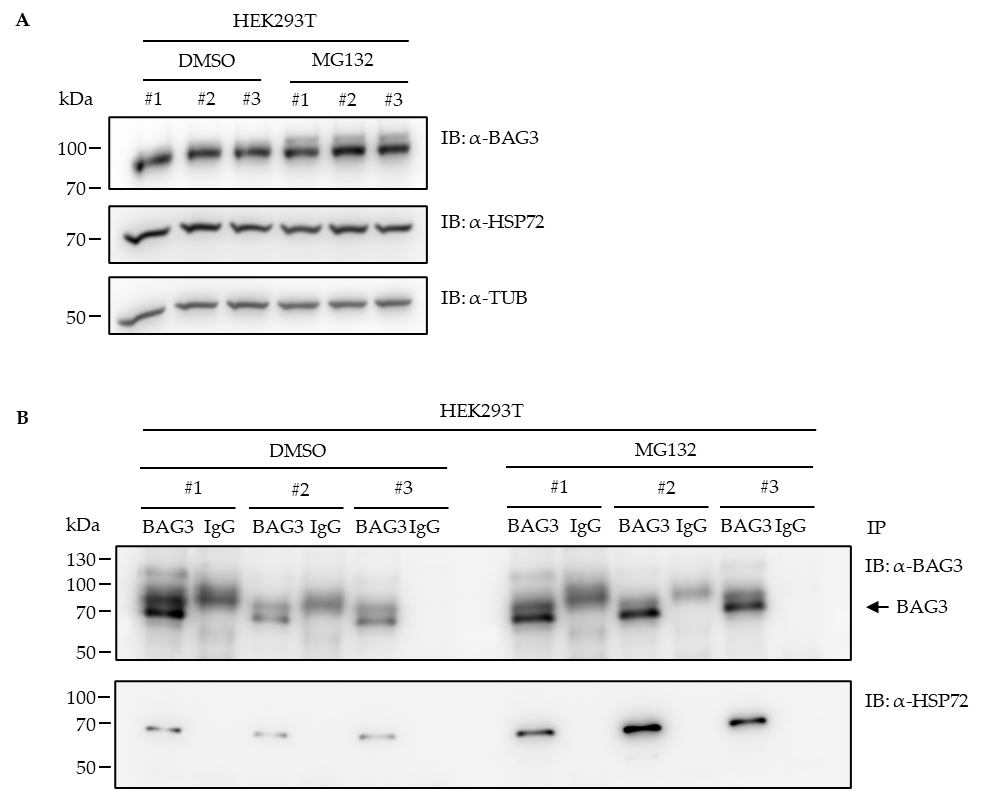


**Figure S2.** Validation of performed affinity purification of endogenous BAG3 performed in qAP-MS approach. HEK293T cells were treated either with DMSO or 10 µM MG132 for 6 h. After extraction of proteins, endogenous BAG3 was immunoprecipitated. As IP control, immunoprecipitation was conducted only with IgG antibodies. Treatment and co-immunoprecipitation assay were performed in three independent biological replicates labelled as #1-#3. (**A**) Inputs of respective co-immunoprecipitation assays #1-#3 were analyzed by immunoblotting. Thereby, expression of endogenous BAG3 and its known interactor HSP72 was checked. Tubulin was used as loading control (**B**) Aliquots of IP eluates (#1-#3) were analyzed by immunoblotting to validate the co-immunoprecipitation assay. BAG3 and its known interactor partner HSP72 could be only detected in BAG3 IP samples, indicating for a good quality of co-immunoprecipitation.
