# Supplementary figures and images for: BAG3 Proteomic Signature under Proteostasis Stress
Source: Cells. 2020 Nov 4;9(11):2416. doi: 10.3390/cells9112416 (PMC7694386; doi:10.3390/cells9112416)

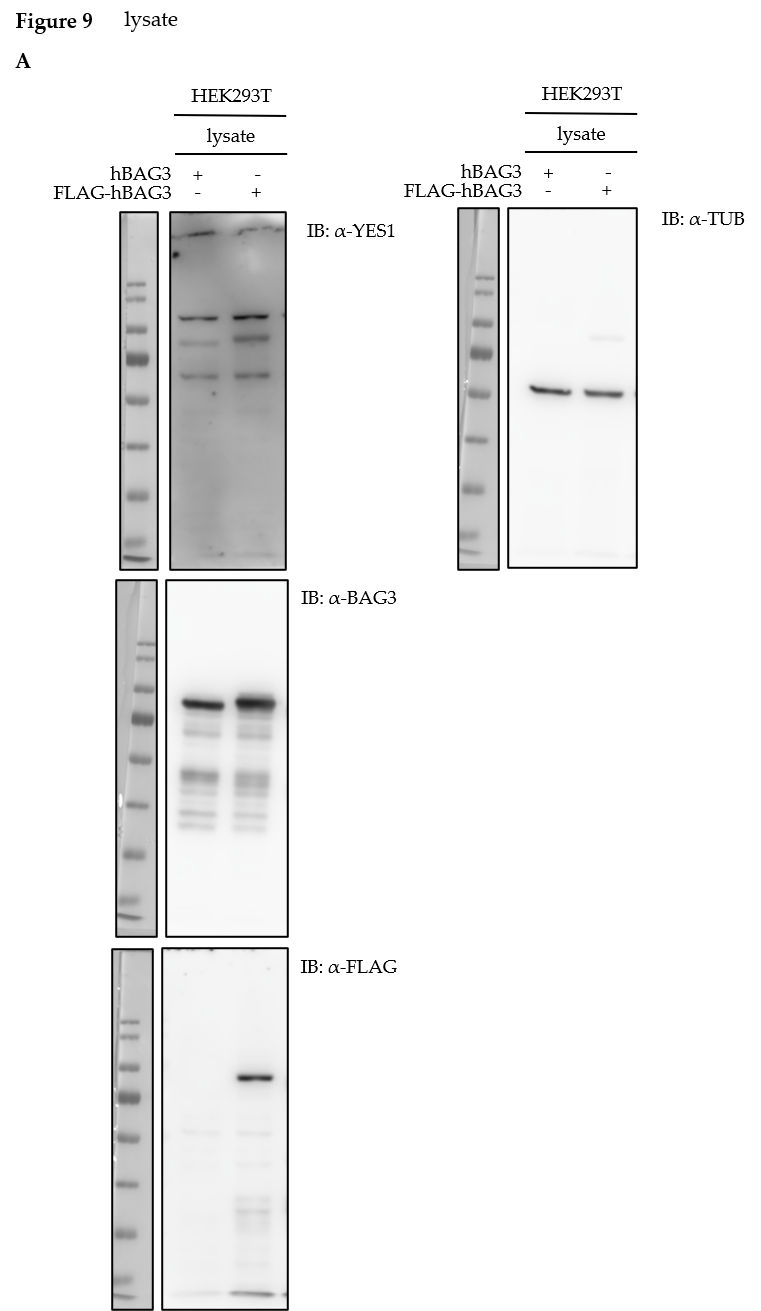


**KD**

**17-**

**26-**

**34-**

**43-**

**55-**

**72-**

**95-**

**130-**

**170-**


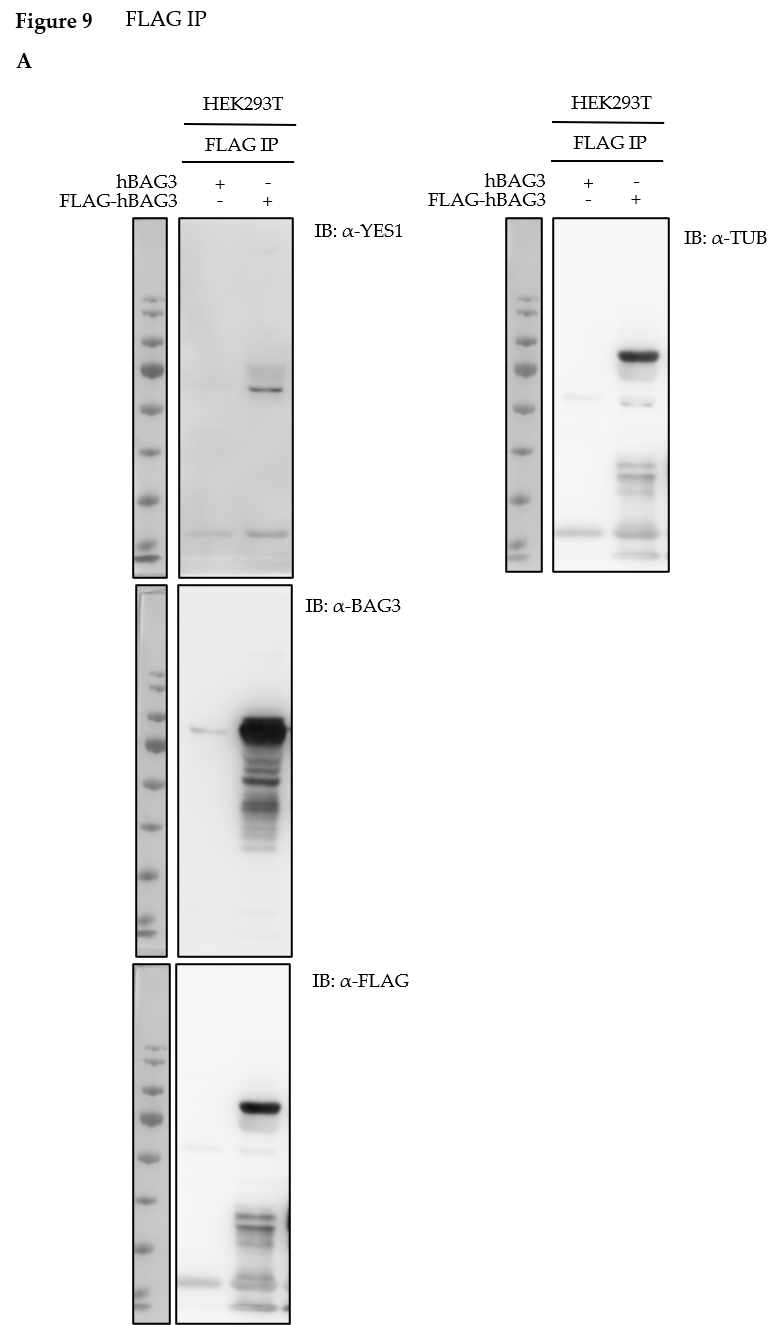


**17-**

**26-**

**34-**

**43-**

**55-**

**72-**

**95-**

**130-**

**170-**

**KD**

Supplement: Supplementary file 1 [file cells-09-02416-s001.zip › Supplements/Full lane Blots Figure 9.docx]
